# CRISPR/Cas9 targeted inactivation of the kauniolide synthase in chicory results in accumulation of costunolide and its conjugates in taproots

**DOI:** 10.3389/fpls.2022.940003

**Published:** 2022-08-29

**Authors:** Katarina Cankar, Johanna Christina Hakkert, Robert Sevenier, Eva Campo, Bert Schipper, Christina Papastolopoulou, Khabat Vahabi, Alain Tissier, Paul Bundock, Dirk Bosch

**Affiliations:** ^1^Wageningen Plant Research, Wageningen University and Research, Wageningen, Netherlands; ^2^Keygene N.V., Wageningen, Netherlands; ^3^Department of Cell and Metabolic Biology, Leibniz Institute of Plant Biochemistry, Halle (Saale), Germany

**Keywords:** chicory, sesquiterpene lactone, kauniolide synthase, genome editing, CRISPR/Cas9, protoplast, costunolide

## Abstract

Chicory taproots accumulate sesquiterpene lactones lactucin, lactucopicrin, and 8-deoxylactucin, predominantly in their oxalated forms. The biosynthetic pathway for chicory sesquiterpene lactones has only partly been elucidated; the enzymes that convert farnesyl pyrophosphate to costunolide have been described. The next biosynthetic step of the conversion of costunolide to the tricyclic structure, guaianolide kauniolide, has so far not been elucidated in chicory. In this work three putative kauniolide synthase genes were identified in chicory named CiKLS1, CiKLS2, and CiKLS3. Their activity to convert costunolide to kauniolide was demonstrated *in vitro* using yeast microsome assays. Next, introduction of CRISPR/Cas9 reagents into chicory protoplasts was used to inactivate multiple chicory KLS genes and several chicory lines were successfully regenerated. The inactivation of the kauniolide synthase genes in chicory by the CRISPR/Cas9 approach resulted in interruption of the sesquiterpene lactone biosynthesis in chicory leaves and taproots. In chicory taproots, but not in leaves, accumulation of costunolide and its conjugates was observed to high levels, namely 1.5 mg/g FW. These results confirmed that all three genes contribute to STL accumulation, albeit to different extent. These observations demonstrate that three genes oriented in tandem on the chicory genome encode kauniolide synthases that initiate the conversion of costunolide toward the sesquiterpene lactones in chicory.

## Introduction

Root chicory (*Cichorium intybus* var. *sativum*) is currently cultivated for the production of inulin, a fructose polymer. Inulin is used as a prebiotic dietary fiber and low calorie sweetener in a variety of food products (Roberfroid, 2002, 2007). Besides inulin, chicory taproots also accumulate sesquiterpene lactones (STLs). Sesquiterpene lactones comprise a class of over 5000 structures of C15 terpenoids with the characteristic lactone ring, that are predominantly produced by plants from the Asteraceae family (Chadwick et al., 2013). STLs show a defense role in plants (Prasifka et al., 2015; Huber et al., 2016; Padilla-Gonzalez et al., 2016) and have been shown to have a variety of bioactivities such as anti-inflammatory, anti-cancer, and analgesic activity (Zhang et al., 2005; Wesolowska et al., 2006; Matos et al., 2021). The STLs in chicory accumulate in laticifers, which are closely associated with the vascular tissues. The compositions of chicory latex has been studied previously and lactucin 15-oxalate, lactucin, 8-deoxylactucin 15-oxalate, and lactucopicrin 15-oxalate were detected as major constituents (Sessa et al., 2000). The STL oxalates are unstable and may on decomposition lead to the accumulation of oxalic acid in the latex released by leaf damage which may contribute to the sensory and antifeedant properties (Sessa et al., 2000).

**FIGURE 1:**
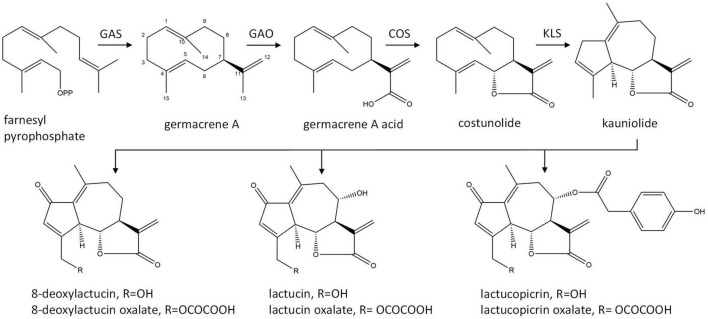
Biosynthetic pathway of chicory sesquiterpene lactones. GAS, germacrene A synthase; GAO, germacrene A oxidase; COS, costunolide synthase; KLS, kauniolide synthase.

The major STLs of chicory belong to the class of guaianolide sesquiterpene lactones and the initial steps in their biosynthesis have been elucidated (Figure 1). The first dedicated step in the biosynthesis of STLs is the conversion of the common sesquiterpene precursor farnesyl pyrophosphate (FPP) to germacrene A by germacrene A synthase (GAS) (Bouwmeester et al., 2002). Four copies of the GAS gene were found in the chicory genome (Bogdanović et al., 2019). Germacrene A is then converted by cytochrome P450 enzymes germacrene A oxidase (GAO) and costunolide synthase (COS) to costunolide (Nguyen et al., 2010; Cankar et al., 2011; Ikezawa et al., 2011; Liu et al., 2011). Although costunolide is an intermediate in the biosynthesis of chicory sesquiterpenes, it does not accumulate in chicory tissues since it is efficiently converted into downstream STLs. The enzyme(s) that are downstream from costunolide and further convert costunolide to STLs remain unknown. These enzymes are probably cytochrome P450 oxygenases of the CYP71 clan, as was shown for the kauniolide synthase (TpKLS) involved in the biosynthesis of guaianolides in feverfew, *Tanacetum parthenium* (Liu et al., 2018). TpKLS converts costunolide to kauniolide and this reaction is also expected to take place during STL biosynthesis in chicory. TpKLS has a unique activity that combines stereoselective hydroxylation of costunolide at the C3 position to form 3α-hydroxycostunolide, followed by additional steps of water elimination, cyclization and regioselective deprotonation. During this multi-step conversion catalyzed by the TpKLS the germacranolide costunolide is converted to the specific tricyclic structure, typical of guaianolides.

Costunolide is a natural sesquiterpene lactone that was first isolated from costus (*Saussurea lappa*) root and later from various other plant species (Rao Vadaparthi et al., 2015). Costunolide has been mostly investigated due to its anti-cancer activity (Li et al., 2020). Its therapeutic potential for diverse other bioactivities has recently been reviewed by Kim and Choi (2019). The bioactivity of costunolide is mediated through its functional moiety, α-methylene-γ-lactone, which can react with the cysteine sulfhydryl group of various proteins. Costunolide is also a common precursor of the germacranolide, eudesmanolide, and guaianolide classes of sesquiterpene lactones that accumulate as bitter tasting compounds in Asteraceae.

Efficient genome editing approaches using CRISPR/Cas9 have been developed for chicory and efficient protocols for subsequent plant regeneration have been described (Bernard et al., 2019; De Bruyn et al., 2020). Transient transfection of either plasmid encoded CRISPR/Cas9 reagents or ribonucleoprotein complexes was used successfully to modulate terpene biosynthesis in chicory (Cankar et al., 2021). Upon inactivation of the germacrene A synthases that catalyze the first dedicated step in the biosynthesis of chicory sesquiterpenes, nearly complete elimination of STLs in chicory was demonstrated (Cankar et al., 2021).

In this study we identified three genes in chicory encoding a putative kauniolide synthase. The strongest expression of the KLS genes was observed in the latex of the chicory taproot. We have demonstrated that all three genes indeed have kauniolide synthase activity in yeast microsomal assays. Inactivation of the kauniolide synthase genes in chicory by CRISPR/Cas9 based genome editing resulted in the interruption of the biosynthesis of chicory sesquiterpenes and the accumulation of costunolide and its conjugates in chicory taproots, compounds with potentially interesting bioactivities that normally do not accumulate in chicory.

## Materials and methods

### Identification of kauniolide synthase and phylogenetic analysis

Three candidate cytochrome P450 genes were identified in the chicory genome by sequence homology to TpKLS and named CiKLS1, CiKLS2, and CiKLS3. The genomic sequence of the KLS locus has been submitted to GeneBank under the accession number ON456175. The protein sequences were submitted for classification into CYP families to dr. David Nelson Nelson (2009) and were assigned the names CYP71BZ25, CYP71BZ26, and CYP71BZ27, respectively. Multiple protein sequence alignments were performed using CLC genomics workbench 20.0.4 software (Qiagen). An unrooted phylogenetic tree was constructed to compare the protein sequence of CiKLS1, CiKLS2, and CiKLS3 to other cytochrome P450 enzymes described to catalyze hydroxylation of germacrene A, germacrene A acid, or costunolide, namely *Lactuca sativa* germacrene A oxidase (LsGAO) (Nguyen et al., 2010), *Cichorium intybus* germacrene A oxidase (CiGAO) (Nguyen et al., 2010), *Lactuca sativa* costunolide synthase (LsCOS) (Ikezawa et al., 2011), *Cichorium intybus* costunolide synthase (CiCOS) (Liu et al., 2011), *Helianthus annuus* germacrene A acid 8-βhydroxylase (HaG8H) (Frey et al., 2018), *Inula hupehensis* germacrene A acid 8β-hydroxylase (IhG8H) (Gou et al., 2018), *Tanacetum parthenium* kauniolide synthase (TpKLS) (Liu et al., 2018), *Tanacetum parthenium* parthenolide synthase (TpPTS) (Liu et al., 2014), *Tanacetum parthenium* 3-βhydroxylase (Tp3BH) (Liu et al., 2014), *Helianthus annuus* eupatolide synthase (HaES) (Frey et al., 2018), and *Helianthus annuus* costunolide 14-hydroxylase (HaC14H) (Frey et al., 2019). Bootstrap N-J trees were generated with a 100 replicates of bootstrap analysis in CLC genomics workbench 20.0.4 software (Qiagen).

### Gene expression analysis

Gene expression of CiKLS1, CiKLS2, and CiKLS3 was studied in a dataset of Illumina pair-based read data of different chicory taproot tissues including cortex, hypodermis, phloem, vascular cylinder, laticifer, and latex tissues, sampled in triplicate (NCBI BioProject PRJNA824299). The Illumina reads were mapped to the genomic sequences of CiKLS1, CiKLS2, and CiKLS3 using CLC genomics workbench 20.0.4 (Qiagen). The paired reads were each counted as one mapped read. The normalized gene expression per each tissue was calculated as reads per kilo base per million mapped reads (RPKM).

### Cloning of candidate genes from chicory for expression in yeast

The coding sequence (CDS) of the chicory kauniolide synthase genes was amplified by PCR from chicory taproot cDNA (Cankar et al., 2011). Next, *Not*I and *Pac*I restriction sites were introduced. The CDS was subsequently cloned into a yeast expression vector pYEDP60 which was modified to contain *Not*I and *Pac*I restriction sites at the polylinker (Pompon et al., 1996; Cankar et al., 2014). Previously characterized TpKLS (Liu et al., 2018) was cloned by the same approach. pYEDP60 plasmids containing CiKLS1, CiKLS2, CiKLS3, TpKLS, and the empty vector pYEDP60 were transformed into yeast strain WAT11 (Urban et al., 1997) using standard procedures (Gietz and Woods, 2002). The recombinant yeast colonies were selected on solid synthetic dextrose minimal medium (SD medium: 0.67% Difco yeast nitrogen base medium without amino acids, 2% d-glucose, 2% agar) supplemented with amino acids, but omitting uracil and adenine sulfate for auxotrophic selection.

### Yeast microsome isolation and *in vitro* enzyme assay

Microsomes from yeast cultures were prepared as previously published (Pompon et al., 1996; Liu et al., 2018). Microsomal preparations from WAT11 cultures containing empty pYEDP60 plasmid were used as negative controls. Enzyme assays were conducted in a total volume of 500 μL, containing 40 mM KPi buffer (pH = 7.5), 200 μM costunolide (Merck life science), 2% DMSO, 2 mM NADPH and 100 μL of microsomal preparation. The enzymatic reactions were incubated for 2 h at 25°C at 250 rpm. For cysteine conjugation 3 mM of cysteine was added and the reactions were incubated at 25°C at 250 rpm for an additional period of 30 min. The reactions were stopped by the addition of an equal volume of methanol containing 0.1% formic acid, the samples were then vortexed, sonicated for 15 min and centrifuged at 21000 g at room temperature. The clear supernatant was transferred to a fresh vial and used for LC-Orbitrap-FTMS analysis. LC-Orbitrap-FTMS analysis was performed as described below.

### CRISPR/Cas reagent design

Three guide RNAs (sgRNAs) targeting different positions in exon 1 of genes CiKLS1, CiKLS2, and CiKLS3 in combination with the *Arabidopsis thaliana* U6 promoter were synthesized and cloned into the pUC57 plasmids (Genscript) (Supplementary Table 1). The target sites of the three guide RNAs were located close to each other within a 90 bp region to enable efficient genotyping. Guides KLSg2 and KLSg3 were targeting genes CiKLS1 and CiKLS2, while guide KLSg21 targeted all three genes without mismatches (Supplementary Figure 3). For SpCas9 expression, a plasmid was made carrying the *A. thaliana* codon-optimized SpCas9 ORF driven by the constitutive parsley ubiquitin promoter in pICH47742 vector backbone (Weber et al., 2011) (plCH47742-pL2-p35S:aCas9:tNOS). For the expression of the green fluorescent protein plasmid pICH47751-pL3-p2×35S:tGFP:t35S was used which was kindly provided by Vera Veltkamp and Ruud de Maagd from Wageningen University and Research. For the RNP approach the Cas9 protein (EnGen Spy-NLS) was obtained from New England Biolabs and the KLSp1 guide was produced using the EnGen sgRNA synthesis kit (E3322V) following the manufacturer’s instructions.

### Isolation and transfection of chicory protoplasts

*Cichorium intybus* subsp. *intybus* var. *sativum* cultivar Orchies was used in the study. *In vitro* clone C37 was selected due to high transformation and regeneration capacity. Chicory leaf protoplasts were prepared by the protocol of Deryckere et al. (2012) with modifications. Chicory plants were grown under sterile conditions on MS medium containing 30 g/l sucrose and 8 g/l micro agar (pH 5.8) at 25°C, with a 16 h/8 h (light/dark) photoperiod at 80 μmol m^–2^ s^–1^ photosynthetic active radiation. For the protoplast isolation leaves of 4–6-week old plants were used. Leaves without midribs (0.5 g) were cut in small pieces of 2 × 2 mm in 1 ml of P0 medium (Deryckere et al., 2012) and pre-incubated for 1 h in 10 ml P0 medium. Next the P0 medium was replaced by 20 ml enzymatic mixture containing 10 g/L cellulase Onozuka R10 (Duchefa Biochemie, Haarlem, Netherlands) and 5 g/L Macerozyme R10 (Duchefa Biochemie, Haarlem, Netherlands). The leaf tissues were incubated overnight in the dark at 25°C; the last hour with gentle agitation at 25 rpm. Next, protoplasts were purified through a 90 μm pore size sieve and pelleted by centrifugation at 100 g for 8 min. The protoplasts were washed with P0 buffer, pelleted and resuspended in 2 mL of buffer P0. Protoplasts (1 ml) were placed on top of 9 ml filter-sterilized 21% sucrose solution and centrifuged at 100 *g* for 10 min. Protoplasts floating on the sucrose cushion were collected and washed with MMG 50 medium (Yoo et al., 2007) (7.3% mannitol, 50 mM MgCl_2_, 4 mM MES, pH 5.7). Protoplasts were then resuspended in 2 ml MMG 50 medium and their concentration was determined using a Bürker counting chamber and adjusted to 1.2 × 10^6^ protoplasts ml^–1^.

For each transfection a mix of 90 μg of the Cas9 expression plasmid and 30 μg of a plasmid carrying sgRNAs was prepared in a 50 μl final volume. For the RNP approach the protocol has been previously described (Cankar et al., 2021). The plasmid or RNPs were added to 300 μl of protoplast solution and mixed gently. Next, 350 μl of PEG-calcium transfection medium (3.6% mannitol, 30% m/v PEG4000, 100 mM CaCl_2_) was slowly added and the transfection mixture was incubated at room temperature for 15 min. The transfection was stopped by slow addition of Medium H (10% mannitol, 1 mM CaCl_2_). The protoplasts were pelleted and resuspended in 2.4 mL medium H at final density of 1.5 × 10^5^ protoplasts/mL. For embedding of protoplasts in alginate (Hall et al., 1996) an equal volume of medium I (10% mannitol, 1 mM CaCl_2_, 2% Na-alginate) was added and aliquots of 1 mL were transferred to a Petri dish containing 10 ml of medium J (Ca-agar plates; 8,25% mannitol, 50 mM CaCl_2_, 0.9% daishin agar, pH 5.9) and left for 1 h to polymerize. The alginate disks were next transferred to tissue culture grade dish containing 4 ml of MC1 medium (Deryckere et al., 2012) and incubated in the dark at 25°C.

After 7 days the MC1 medium was replaced by MC2 medium (Deryckere et al., 2012) and light intensity was increased to 40 μmol m^–2^ s^–1^ and the medium was refreshed once after 10 days. After 4 weeks the alginate layer was cut into strips and placed onto MC2-agarose medium [MC2 medium (Deryckere et al., 2012) without mannitol, containing 20 g/L sucrose and 8 g/L agarose] to stimulate callus growth. In week 7 individual calli were transferred to MC3-A medium [MC3 medium (Deryckere et al., 2012), supplemented with 0.5 mg/L BAP, 0.5 mg/L IAA and 6 g/L micro agar] and 4 weeks later to MC3-B medium [MC3 medium (Deryckere et al., 2012), supplemented with 0.25 mg/L BAP, 0.25 mg/L IAA and 6 g/L micro agar] to induce shoot regeneration. Callus was transferred to fresh medium every 3–4-weeks. Regenerated shoots were placed on solid MS medium containing 20 g/l sucrose and 8 g/l micro agar (pH 5.8). Three clones of 9 genome edited lines were transferred to soil and grown for a period of 3 months at 20°C with a 16 h/8 h (light/dark) photoperiod to form a taproot.

### Genotyping chicory plants

To assess the efficiency of genotyping at an early stage a PCR was performed on a population of approximately 36,000 protoplasts 48 h after transfection using the Phire Plant Direct PCR Kit (Thermo Fischer) amplifying the exon 1 region of CiKLS1, CiKLS2, and CiKLS3. A nested PCR was done on each PCR product using common forward and reverse primers for all three genes (Supplementary Table 1) and a final third PCR was performed with barcoded Illumina primers to enable later identification of the sequences. All of these PCR products were then pooled and paired-end sequenced on an Illumina MiSeq apparatus. The sequences were analyzed for the presence of indel mutations at the target sites.

The genotyping of calli and shoots was performed by Sanger sequencing. A PCR product was amplified from a small sample of the tissue using Phire Plant Direct PCR Kit (Thermo Fischer) using common primers targeting genes CiKLS1, CiKLS2, and CiKLS3. The PCR products were sent for Sanger sequencing and analyzed for the presence of indels at the target sites.

For detailed genotyping of nine mutant lines showing large differences in the terpene profile specific PCR products for genes CiKLS1, CiKLS2, and CiKLS3 were amplified by Phire Plant Direct PCR Kit (Thermo Fischer) and PCR products were subsequently purified and cloned into the pJET1.2 vector (Thermo Fischer) and sequenced by Sanger sequencing. Sequence data was analyzed by SeqMan Pro software (DNASTAR). Cas9 gene integration was checked by PCR using specific primers that amplified an 800 bp fragment of the Cas9 gene (Supplementary Table 1).

### Analysis of sesquiterpene lactones by LC-Orbitrap-FTMS

Edited and WT chicory leaf and taproot material was frozen and powdered in liquid nitrogen. Extraction from 300 mg of tissue was performed using methanol containing 0.1% formic acid, the samples were then vortexed, sonicated for 15 min and centrifuged at 21000 *g* at room temperature. The clear supernatant was transferred to a fresh vial and used for LC-MS analysis. LC-MS analysis was performed using a LC-PDA-LTQ-Orbitrap FTMS system (Thermo Scientific), which consists of an Acquity UPLC (H-Class) with Acquity elambda photodiode array detector (220–600 nm) connected to a LTQ/Orbitrap XL hybrid mass spectrometer equipped with an electrospray ionizator (ESI). The injection volume was 5 μl. Chromatographic separation was on a reversed phase column (Luna C18/2,3 μ, 2.0 × 150 mm; Phenomenex, United States) at 40°C. Degassed eluent A [ultra-pure water: formic acid (1000:1, v/v)] and eluent B [acetonitrile:formic acid (1000:1, v/v)] were used at a flow rate of 0.19 ml min^–1^. A linear gradient from 5 to 75% acetonitrile (v/v) in 45 min was applied, which was followed by 15 min of washing and equilibration. FTMS full scans (m/z 90.00–1350.00) were recorded with a resolution of 60,000 FWHM. The free costunolide content and the total amount of costunolide and its conjugates was quantified by comparing the peak area of the corresponding peaks in the PDA chromatogram at 280 nm to the standard curve of costunolide standards (Merck life science).

### GC-MS analysis of chicory taproot and leaf extracts

Edited and WT chicory root and leaf material (300 mg) were frozen and powdered in liquid N_2_. The samples were extracted with 1.5 mL of hexane: ethyl acetate mixture (v/v 85:15). The extracts were sonicated for 15 min and centrifuged for 10 min at 1200 *g*. The extracts were dried over a Na_2_SO_4_ column prepared in a glass wool plugged glass pipette. Analytes from 1 μL samples were separated using a gas chromatograph (5890 series II; Hewlett-Packard) equipped with a 30 m × 0.25 mm, 0.25 mm film thickness column (ZB-5; Phenomenex) using helium as carrier gas at flow rate of 1 mL/min. The injector was used in splitless mode with the inlet temperature set to 250°C. The initial oven temperature of 45°C was increased after 1 min to 310°C at a rate of 10°C/min and held for 5 min at 310°C. The GC was coupled to a mass-selective detector (model 5972A; Hewlett-Packard), scanning from 45 to 500 atomic mass units.

## Results

### Identification of the genes encoding chicory kauniolide synthase

To identify cytochrome P450 genes that are involved in the kauniolide biosynthesis in chicory, the chicory genome was queried to identify homologues of the *Tanacetum parthenium* kauniolide synthase gene (TpKLS). Three candidate genes that encode a protein of 497 amino acids were identified in the chicory genome. The three encoded enzymes showed 62% amino acid identity and 72% nucleotide sequence identity to the TpKLS (Supplementary Figure 1). The chicory KLS genes cluster together with the TpKLS in the phylogenetic tree when compared with other CYP71 enzymes which were described to oxygenate either germacrene A, germacrene A acid, and/or costunolide in Asteraceae family (Supplementary Figure 1). The amino acid sequences of the three TpKLS homologues were highly similar to each other showing 98–99% amino acid identity (Supplementary Figure 1). The nucleotide sequences showed 96–99% nucleotide identity. These genes were named *Cichorium intybus* kauniolide synthase 1, 2, and 3 (CiKLS1, CiKLS2, and CiKLS3). All three genes were classified into the CYP71 family of cytochrome P450 enzymes (Nelson, 2009) and were assigned CYP names CYP71BZ25, CYP71BZ26, and CYP71BZ27, respectively. The alignment of the CiKLS genes with TpKLS and the analysis of conserved domains is shown in Supplementary Figure 1. The open reading frames of all three genes consisted of two exons and one intron. The genes were located tandemly on a 40 kB region showing that the genes are physically linked in the chicory genome (Supplementary Figure 2).

### Gene expression of CiKLS genes in chicory taproot tissues

**FIGURE 2:**
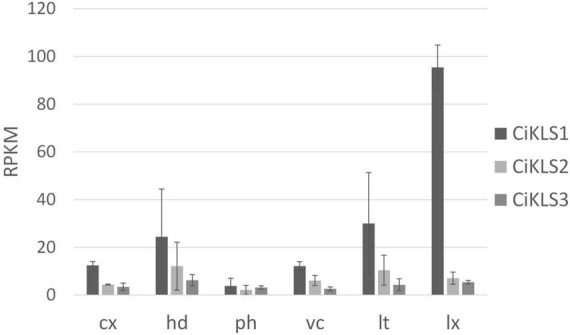
Gene expression of the chicory kauniolide synthase genes CiKLS1, CiKLS2, and CiKLS3 in chicory root tissues. Cx, cortex; hd, hypodermis; ph, phloem; vc, vascular cylinder; lt, laticifer; lx, latex; RPKM, Reads per kilo base per million mapped reads. Mean and standard deviation for three biological replicates are shown.

Gene expression of the CiKLS1, CiKLS2, and CiKLS3 genes was analyzed in a RNAseq dataset comprising of different tissues of chicory taproot. The CiKLS1 gene was found to be particularly highly expressed in the latex and showed lower gene expression in other root tissues. Genes CiKLS2 and CiKLS3 showed low expression in all root tissues (Figure 2). This finding indicated that although the CiKLS1 is the most strongly expressed gene also CiKLS2 and CiKLS3 may partially contribute to the biosynthesis of chicory STLs in taproots.

### Functional characterization of chicory kauniolide synthase in yeast

**FIGURE 3:**
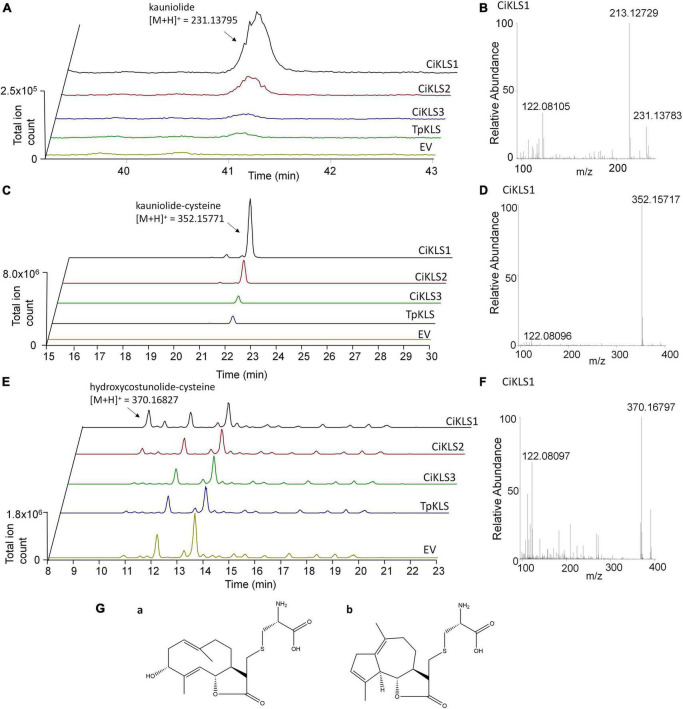
Analysis of *in vitro* enzyme activity of CiKLS enzymes in yeast microsome assays. **(A)** LC-Orbitrap-FTMS chromatograms are shown at m/z = 231.13795 typical for kauniolide (5 ppm, positive ionization mode) for enzyme assay using microsomes expressing CiKLS1, CiKLS2, CiKLS3, and TpKLS incubated with costunolide. Microsomes from a yeast strain containing empty pYEDP60 vector (EV) are used as control. **(B)** Mass spectrum of kauniolide produced by CiKLS1. **(C)** LC-Orbitrap-FTMS chromatograms are shown at m/z = 352.15771 typical for kauniolide-cysteine (5 ppm, positive ionization mode) for enzyme assay using microsomes expressing CiKLS1, CiKLS2, CiKLS3, and TpKLS incubated with costunolide followed by cysteine conjugation. Microsomes from a yeast strain containing empty pYEDP60 vector (EV) are used as control. **(D)** Mass spectrum of kauniolide-cysteine produced by CiKLS1. **(E)** LC-Orbitrap-FTMS chromatograms are shown at m/z = 370.16827 typical for 3α-hydroxycostunolide-cysteine (5 ppm, positive ionization mode) for enzyme assays with CiKLS1, CiKLS2, CiKLS3, and TpKLS incubated with costunolide followed by cysteine conjugation. Microsomes from a yeast strain containing empty pYEDP60 vector (EV) are used as control. **(F)** Mass spectrum of 3α-hydroxycostunolide-cysteine produced by CiKLS1. **(G)** Presumed molecular structure of 3α-hydroxycostunolide-cysteine (a) and kauniolide-cysteine (b).

To functionally characterize the three candidate cytochrome P450 genes the three chicory KLS genes and the TpKLS gene were cloned into the pYEDP60 vector and transformed into the yeast strain WAT11 (Pompon et al., 1996). Gene expression was induced by addition of galactose to the growth medium and then microsomal fractions containing the cytochrome P450 enzymes and the *A. thaliana* cytochrome P450 reductase ATR1 were isolated. The microsomal fractions were used in enzymatic assays in which costunolide was added as substrate. Upon incubation the CiKLS and TpKLS enzymes produced a product with mass [M + H]^+^ = 231.1378 from costunolide as shown by LC-Orbitrap-FTMS (Figure 3). This mass corresponds within 5 ppm accuracy with the mass of kauniolide. However, the peaks in the chromatograms were very small, probably due to the poor ionization efficiency of kauniolide. In a parallel enzyme reaction the assay mixtures were incubated with L-cysteine after the assay to obtain cysteine conjugates of costunolide and kauniolide which are more efficiently detected by LC-MS. Cysteine conjugation occurs non-enzymatically, is irreversible and adds an exact mass of [M] = 121.01464 (Liu et al., 2018). The analysis of conjugated products revealed the formation of two new products for all three chicory KLS genes as well as for the previously characterized TpKLS. These peaks were not observed when costunolide was incubated with microsomes from a WAT11 yeast strain containing an empty pYEDP60 vector. The major product of the enzymatic assay eluted at the retention time of 22.09 min and its accurate mass corresponded within 5 ppm accuracy to the mass of [M + H]^+^ = 352.15771, which is the mass of kauniolide-cysteine (Figure 3). The product co-eluted and matched in accurate mass to kauniolide-cysteine produced by TpKLS. A second minor product was observed at the retention time of 10.58 min and corresponded within 5 ppm accuracy to the mass of [M + H]^+^ = 370.16827 which is the mass for a cysteine conjugate of costunolide with one additional hydroxyl group (Figure 3). As postulated for the formation of kauniolide in *Tanacetum parthenium* (Liu et al., 2018), this is likely to be a hydroxylation of costunolide at the C3 position resulting in the formation of 3α-hydroxycostunolide, which may be an intermediate in kauniolide biosynthesis. These findings confirmed that all three chicory KLS genes encode functional enzymes that have kauniolide synthase activity similar to that of the previously characterized TpKLS.

### CRISPR/Cas9 mutagenesis of the chicory KS genes

Next, inactivation of the chicory kauniolide synthase homologues was performed in chicory leaf mesophyll protoplasts using CRISPR/Cas9. As no selection markers were introduced during the editing procedure, we first assessed the transfection and regeneration efficiency. To this end, the fluorescent reporter plasmid expressing GFP under the control of the 35S promoter was transfected to chicory protoplasts. After 1 day 42 ± 1% of the protoplasts showed GFP signal in duplicate experiments. Protoplast regeneration was evaluated after 7 days and 85 ± 3% of the protoplasts showed cell division. After 15 days 96 ± 0% of the protoplasts showed regeneration. The established protocol had therefore led to high transfection and regeneration efficiencies and was deemed appropriate for genome editing.

In the first approach to genome editing, CRISPR/Cas9 reagents were expressed transiently by transfection of the chicory protoplasts with two plasmids. The first plasmid carried an *A. thaliana* codon-optimized SpCas9 ORF driven by the constitutive parsley ubiquitin promoter. A second plasmid expressed three guide RNAs to maximize the chance of inactivating all three KLS genes, each under the control of the *A. thaliana* U6 promoter. The three guide RNAs were designed to target exon 1 of the three kauniolide synthase genes (Supplementary Figure 3). The guides map closely to each other within a 90 bp region of exon 1 to enable efficient genotyping. Two guides named KLSg2 and KLSg3 were designed to target CiKLS1 and CiKLS2 genes without any mismatches. KLSg2 showed one mismatch and KLSg3 showed two mismatches to CiKLS3. However, SpCas9 is able to tolerate a limited number of mismatches depending on their position. Guide KLSg21 was designed to target all three genes CiKLS1, CiKLS2, and CiKLS3 without mismatches. In the second approach a ribonucleoprotein (RNP) complex was used to inactivate the CiKLS genes. This involved the introduction of the Cas9 protein together with an *in vitro* synthesized guide RNA into chicory mesophyll protoplasts. In this case only a single guide was used (KLSp1) which targets all three CiKLS genes without mismatches (Supplementary Figure 3).

In order to estimate the mutation efficiency at an early experimental stage, a subpopulation of the protoplasts was used for genotyping. For this purpose, protoplasts were harvested 48 h after transfection with the CRISPR/Cas9 constructs or RNPs. Firstly, the entire exon 1 was amplified using specific primers for genes CiKLS1, CiKLS2, and CiKLS3 and a then a nested PCR using common primers was done to amplify a 141 bp region encompassing the target sites of the three guides. The PCR products were then bulk sequenced to quantify the percentage of amplicons for each gene that contained CRISPR/Cas9-mediated mutations. The analysis showed that when the CRISPR/Cas9 reagents were expressed from plasmids, in total an editing efficiency of 8.4, 5.5, and 3.4% was observed for genes CiKLS1, CiKLS2, and CiKLS3, respectively (Table 1). Besides indels at the target sites mostly larger deletions ranging from 38 to 62 bp were observed corresponding to the deletion of the region between guides KLSg2 and KLSg21 or KLSg3 and KLSg21 for genes CiKLS1 and CiKLS2. A lower frequency of editing was observed for gene CiKLS3, probably since this gene was targeted only by guide KLSg21 (Supplementary Figure 4). No genome editing with guide KLSg2 and KLSg3 was observed for this gene indicating high specificity of guides KLSg2 and KLSg3 with a single and double mismatch to CiKLS3, respectively. When RNPs were used to create CiKLS mutations we found that approximately 3% of the sequence reads carried indel mutations.

**TABLE 1:** Summary of genotyping results of the gene edited chicory plants.

Tissue	Number of individuals genotyped	Editing efficiency CiKLS1	Editing efficiency CiKLS2	Editing efficiency CiKLS3	Genotyping approach
**Protoplast**					
Plasmid approach	36,000	8.4%	5.5%	3.4%	Illumina sequencing
RNP approach	36,000	nd	3%	nd	
**Calli (7 weeks)**					
Plasmid approach	30	23% of calli show editing in either of CiKLS genes			Sanger sequencing of a common PCR amplicon
RNP approach	nd				
**Shoots (4 months)**					
Plasmid approach	200	27% of shoots show editing in either of CiKLS genes			Sanger sequencing of a common PCR amplicon
RNP approach	72	6%	9%	11%	Sanger sequencing of a specific PCR amplicon
Regenerated plants*					
Plasmid approach	8	100%	100%	50%	Cloning and Sanger sequencing of a specific PCR amplicon
RNP approach	1	100%	100%	100%	

*Detailed description of the genotypes is given in Supplementary Table 2, nd, not determined.

Expression of CRISPR/Cas9 reagents from plasmid constructs gave a mutation frequency in the protoplast population that was deemed sufficient to proceed with the regeneration of calli. After calli formation in the alginate layer editing efficiency was monitored again. The editing efficiency was assayed using PCR amplification of the exon 1 region with primers targeting all three kauniolide synthase genes. The PCR products were sequenced by Sanger sequencing and the sequences were examined for presence of mutations in the amplified regions. The analysis of 30 calli by this approach revealed that mutations were found in 23% of the analyzed calli. The editing frequency was deemed sufficient to proceed to regeneration of shoots and 500 calli were transferred to regeneration medium 7 weeks after transfection. From these calli, 68% formed shoots. In total 300 shoots were transferred to rooting medium and shoots of 200 chicory lines were analyzed for presence of mutations in any of the kauniolide synthase genes. The analysis showed presence of mutations in exon 1 for 27% of the analyzed plants. Mutations were assayed using PCR amplification of the exon 1 region with primers targeting all three kauniolide synthase genes. By this approach a quick screening and selection of gene edited chicory lines was achieved, though this does not reveal the exact identity of the mutations in each of the six amplified alleles of the kauniolide synthase. Thus, in total 54 chicory shoots were obtained that showed mutations in at least one of the three kauniolide synthase genes. While the introduction of RNPs into protoplasts gave a somewhat lower mutagenesis frequency, this was still thought sufficient to proceed. In this case no genotyping of the calli was performed and 72 independent shoots were regenerated for further analysis. A selection of these lines created by both methods were further characterized with respect to STL accumulation and with respect to their mutations as described below.

### Detailed analysis of mutations in the three kauniolide synthase genes in nine regenerated chicory lines

In total eight chicory lines obtained by the plasmid transfection and one chicory line obtained by the RNP transfection were fully regenerated and genotyped in detail. Firstly, as the Cas9 reagents were introduced into protoplasts *via* a transient Cas9 plasmid approach in eight lines, integration of the Cas9 gene was assayed. The integration of Cas9 in the genome was observed in lines A1, A34, and A153 but not in the other five analyzed lines. The integration of Cas9 has been previously observed when using plasmid-based transfection of CRISPR/Cas9 reagents for chicory genome editing (Bernard et al., 2019; Cankar et al., 2021).

Next, a detailed genotyping was performed on the 8 chicory lines produced using the CRISPR/Cas9 plasmid approach and carrying mutations in the kauniolide synthase genes that were transferred to the greenhouse for formation of taproots. No wild types alleles could be detected for CiKLS1 nor CiKLS2, instead sequencing of the amplified regions suggest homozygous or biallelic mutations in all eight analyzed lines for these two genes (Supplementary Table 2). All three guide RNAs appeared efficient in gene editing as frequently deletions in size between 62 and 72 bp were observed between guides KLSg2 and KLSg21 and deletions in size 38–49 bp were observed between guides KLSg3 and KLSg21. In several cases a small indel at the location of guide KLSg2 was found to be combined with a deletion between guides KLSg3 and KLSg21 for the same allele. Only in two cases a larger insertion of 27 and 114 bp in was observed in CiKLS1 and CiKLS2, respectively. For a number of lines which were classified to have homozygous mutations it is possible that larger rearrangements prevent the amplification of the target region and in that case only one allele was preferentially amplified and sequenced. The CiKLS3 gene which was, in contrast to CiKLS1 and CiKLS2, targeted with only one guide with zero mismatches, was less efficiently mutated. In three plants (A16, A144, A153) only WT alleles of CiKLS3 were observed and line A148 appears heterozygous as a WT allele was detected next to a mutated allele of CiKLS3 (Supplementary Table 2). Nevertheless, in the four other plants both alleles of CiKLS3 appear mutated, and thus no WT alleles of any of the three CiKLS genes could be detected. The regenerated chicory line which was generated using the RNP approach (B72) was also genotyped. In this case a single guide RNA had been used, reducing the chance of any larger rearrangements. All three of the CiKLS genes were also found to be mutated in this line (Supplementary Table 2). In this line only single nucleotide insertions and deletions were observed.

In summary, five chicory lines (A1, A5, A34, A83, and B72) are full kauniolide synthase knockouts as no intact alleles of any of the three CiKLS genes are present. The other four lines also have all alleles of CiKLS1 and CiKLS2 mutated but also carry WT CiKLS3 alleles, one (line A148) or both (lines A16, A144, A153).

### The inactivation of chicory KS genes prevents terpene accumulation in chicory leaves

Although STL biosynthesis in chicory is relevant in its taproots, we decided to examine STL accumulation in developing shoots as a criterium for an early stage selection of plants for further propagation and taproot development in soil. To that purpose, leaves of 45 gene edited chicory shoots growing in tissue culture, which showed mutations in the kauniolide synthase gene, were sampled for metabolite analysis. These 45 chicory lines were chosen for analysis since they were collected early in the process of regenerating shoots and sufficient material was available. None of these shoots showed a visual growth phenotype. From these lines 31 were generated using plasmid transfection and 14 lines were generated by RNP approach. The LC-Orbitrap-FTMS (liquid chromatography-Orbitrap-Fourier transform mass spectrometry) analysis of the leaves revealed that the accumulation of major chicory STLs, namely lactucin, lactucopicrin, and 8-deoxylactucin and their oxalates, was nearly completely eliminated in 24 of the 45 analyzed chicory lines (Supplementary Figure 5), from these 23 lines were generated by the plasmid approach and one line was generated by the RNP approach. This analysis indicated that the kauniolide synthase candidate genes are indeed involved in STL biosynthesis. However, and surprisingly given what is known of STL biosynthesis in chicory taproots, no accumulation of costunolide or other pathway intermediates was observed in the leaves. Next, nine chicory lines showing STL elimination in leaves were selected for detailed analysis. The selection criteria was that the plants carried a mutation in one or more KLS genes and showed nearly complete elimination of terpenes in tissue culture leaves. These nine lines did not differ from other obtained edited lines and did not show a visual phenotype compared to WT plants. Lines were multiplied in tissue culture, and three clones of each line were transferred to soil to investigate STL biosynthesis in taproots.

### The inactivation of chicory KS genes leads to accumulation of costunolide and its conjugates in chicory taproots

**FIGURE 4:**
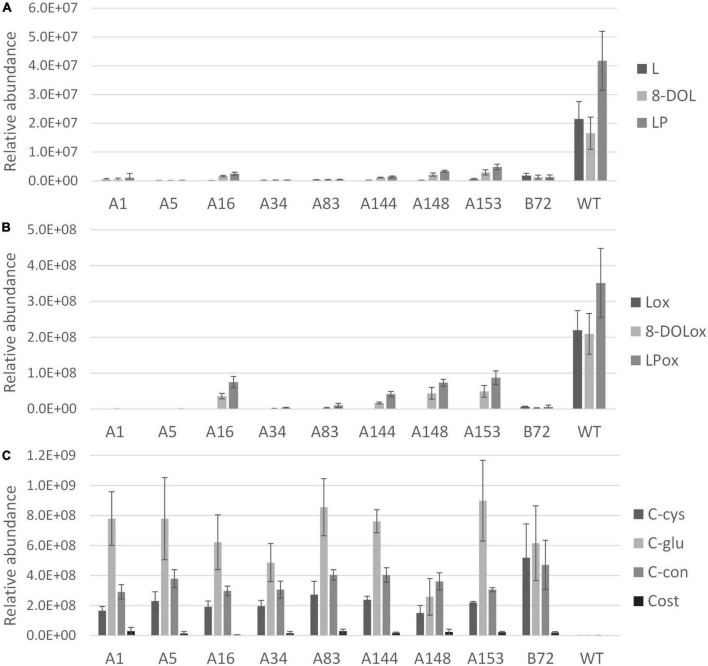
Terpene profile analysis of gene edited chicory lines by LC-Orbitrap-FTMS. Peak area of terpenes in roots of 9 genome edited chicory lines (A1, A5, A16, A34, A83, A144, A148, A153, and B72) were analyzed in triplicate and compared to WT chicory lines (*n* = 11). Panel **(A)** shows reduction of lactucin (L), 8-deoxylactucin (8-DOL), and lactucopicrin (LP) in the gene edited lines. Panel **(B)** shows the reduction of lactucin 15-oxalate (Lox), 8-deoxylactucin 15-oxalate (8-DOLox), and lactucopicrin 15-oxalate (LPox) in the gene edited lines. Panel **(C)** shows the accumulation of costunolide (cost), costunolide-cysteine (C-cys), costunolide-glutathione (C-glu), and a novel costunolide conjugate (C-con) in the gene edited lines.

After 3 months of cultivation in soil, leaves and taproots of nine chicory lines grown in three biological replicates were sampled for terpene profiling. The LC-Orbitrap-FTMS analysis of the leaves showed that major chicory STLs were eliminated, similarly as observed in the tissue culture leaves. In taproots the content of the major chicory STLs and their oxalates was completely eliminated in five lines designated A1, A5, A34, A83, and B72, while in lines A16, A144, A148, and A153 a small amount of STLs was still present in the taproot (Figure 4). Interestingly, all the lines that have residual levels of sesquiterpene lactones still carry WT alleles of CiKSL3, indicating that CiKSL3 despite its low expression level does contribute to the production of STLs.

In contrast to the leaves, accumulation of four new compounds was observed in the taproot tissue (Supplementary Figure 6). Upon inactivation of the kauniolide synthase an increase of its substrate costunolide would be expected. Indeed, accumulation of free costunolide was observed in the nine genome-edited lines while no free costunolide accumulation was observed in WT taproots. The free costunolide amount ranged from 21 ± 6 μg/g in line A16 to 160 ± 76 μg/g FW in line A83. However, costunolide was detected only as a minor terpene product in the genome-edited taproots. Three major peaks in the LC-Orbitrap-FTMS chromatogram were identified as costunolide conjugates. The first compound eluting at the retention time of 19.96 and with an accurate mass corresponding to [M + H]^+^ = 354.17335 with 5 ppm accuracy was identified as costunolide-cysteine. The compound eluting at the retention time of 19.97 had the accurate mass that corresponded to the mass of [M + H]^+^ = 540.23741 with 5 ppm accuracy and was identified as costunolide-glutathione. The formation of costunolide-cysteine and costunolide-glutathione has previously been shown in *Nicotiana benthamiana* plants that were transiently expressing the chicory STL biosynthetic pathway to produce costunolide (Liu et al., 2011). Additionally, a new conjugate with the mass of [M + H]^+^ = 441.15692 was identified, which was not previously described in other plants. This mass corresponds to the mass of costunolide with an additional mass of 208.00375. The total amount of costunolide (free and conjugated) was estimated based on the absorbance at 280 nm. In all genome-edited lines costunolide and its conjugates accumulated to high levels and on average 1.5 ± 0.54 mg/g FW total costunolide products accumulated in the taproots.

Additionally GC-MS analysis of chicory taproot extracts was performed to study whether other apolar intermediates of the STL biosynthesis in chicory may have accumulated in the chicory taproot. Extracts of the taproots of the nine chicory lines were analyzed by GC-MS and in none of the extracts accumulation of germacrene A was observed. Besides costunolide, accumulation of germacrene A acid was observed (Supplementary Figure 7).

## Discussion

In this work we show that kauniolide synthase in chicory is encoded by three functional ORFs which are located in tandem arrangement on the chromosome. The encoded chicory enzymes only show 62% amino acid identity to the previously characterized TpKLS. However, the chicory and the *Tanacetum parthenium* KLS enzymes cluster closely together when compared with other cytochrome P450 enzymes of the CYP71 clan that have been shown to hydroxylate either germacrene A, germacrene A acid, or costunolide (Supplementary Figure 1). Interestingly, KLS genes cluster closely with the costunolide synthase genes and germacrene A acid 8-hydroxylase genes, that use germacrene A acid as a substrate, rather than with enzymes that, like KLS, use costunolide as a substrate, such as the parthenolide synthase, and the costunolide 14-hydroxylase. This suggests that as in *Tanacetum parthenium* the chicory KLS enzymes evolved out of COS. Blast analysis of the NCBI nucleotide collection (nr/nt) and NCBI expressed sequence tag database (EST) revealed that close homologues showing larger than 90% protein sequence identity to the chicory KLS can be found in *Lactuca sativa* and *Lactuca virosa* species, which are also both known to produce high amount of guaianolide STLs (Sessa et al., 2000).

*In vitro* activity analysis of the three KLS enzymes expressed in yeast shows that all three genes show kauniolide synthase activity. Detection of a 3α-hydroxycostunolide as minor product indicates that kauniolide formation proceeds *via* C3 hydroxylation as previously postulated for the kauniolide synthase of *Tanacetum parthenium*. The gene expression analysis shows that CiKLS1 has the highest expression in the chicory root tissues and may therefore contribute the most to the biosynthesis of STLs in chicory taproot. Furthermore, CiKLS1 has the highest activity in the *in vitro* microsome assays but this may also be due to higher expression or better folding of this enzyme in yeast. Additionally, the activity of CiKLS enzymes in yeast may be improved further by co-expression of the chicory cytochrome P450 reductase instead of the *A. thaliana* ATR1.

Upon inactivation of the three genes in chicory, complete elimination of STLs was observed when all six alleles of the CiKLS gene were mutated. A small amount of STLs are still synthesized in chicory lines where only one or two WT alleles of CiKLS3 are present showing that this lowly expressed gene also contributes to STL formation in taproots. Both *in vitro* analysis and the analysis of mutant plants therefore indicate that while CiKLS1 may have the highest expression in the taproot, all three chicory KLS enzymes have kauniolide synthase activity and may contribute to STL biosynthesis in chicory. The three chicory KLS genes are highly homologous and also closely located in tandem orientation in the chicory genome which indicates that they have been generated by recent duplication events. Similarly, several copies of the GAS gene that catalyzes the first dedicated step in STL biosynthesis have been found in the chicory genome (Bogdanović et al., 2019).

Germacrene A synthase promoter activity analysis indicated that the initial step in the chicory STL biosynthesis, namely the germacrene A synthesis, may be active in all cell types of the chicory root. The highest expression of the GAS genes was found in the outer layer of the root (Bogdanović et al., 2019). In contrast the kauniolide synthase gene shows the highest gene expression in the latex suggesting that its substrate costunolide is also present in the latex. This indicates that chicory laticifers are not only important for STL storage but that the final steps of STL biosynthesis are actually located in the latex. This spatial separation of STLs in the latex may be needed due to their high reactivity.

Upon inactivation of the kauniolide synthase in chicory taproots its substrate costunolide accumulates. The plants showed no visual phenotypic difference compared to the WT plants under the cultivation conditions used. In taproots there appears to be no other biosynthetic pathway that can metabolize costunolide and convert it to other sesquiterpene lactones in chicory. Although a small amount of free costunolide was detected in chicory taproot the majority of costunolide was found to be conjugated to cysteine, glutathione and an unidentified compound. The observed conjugates might be related to the reactivity of the α-methylene-γ-lactone domain of costunolide (Kim and Choi, 2019), which is normally rapidly converted in the latex by KLS but now becomes conjugated. The conjugation may be catalyzed enzymatically but was also shown to occur non-enzymatically upon incubation of costunolide with cysteine and glutathione. Formation of conjugates has also been observed when the costunolide biosynthetic pathway was heterologously expressed in *Nicotiana benthamiana* (Liu et al., 2011). An additional effect of the conjugation is that the solubility is increased. While costunolide is an intermediate in the biosynthesis of several types of sesquiterpene lactones, it rarely accumulates in the free form. The costus plant is indigenous to India and Pakistan, where it grows in the Himalayas at 2500–3500 m elevations and its roots are used for medicinal purposes. In this plant costunolide and dehydrocostus lactone are the major components and seem to accumulate efficiently in the free form in costus oil (Rao Vadaparthi et al., 2015). Perhaps in costus the conjugation of costunolide due to its high reactivity is mitigated by sequestering it to the oil.

CRISPR/Cas9 mutagenesis is shown as an efficient strategy to inactivate genes in chicory. In this work we transiently expressed CRISPR/Cas9 reagents encoded on plasmids in protoplasts and as observed previously, transient plasmid protocols can result in Cas9 integration in the chicory genome despite the absence of selection (Bernard et al., 2019; Cankar et al., 2021). Through the transient transfection of a ribonucleoprotein complex we were able to identify CiKLS mutants that lack additional insertions. The CRISPR/Cas9 mutagenesis has been an efficient tool to elucidate enzyme function, to contribute to the knowledge on which gene copy contributes most to the biosynthesis in case of multi-gene families and is an efficient strategy to create useful novel biochemical phenotypes. Chicory taproots can accumulate high amount of terpenes, which are currently unused. Here we describe a strategy to accumulate costunolide and its conjugates to high levels in an industrial crop. Combined levels of conjugates and free costunolide correspond to 1.5 mg/g FW opening possibilities to use chicory as a production platform for costunolide.

## Data availability statement

The data presented in this study are deposited in the NCBI repository under accession numbers ON456175 and PRJNA824299.

## Author contributions

KC and DB devised the study. JH, KC, PB, RS, BS, and EC performed the experiments and analyzed the data. CP performed the bioinformatics analysis. KV and AT provided RNA-Seq data for gene expression analysis. KC, JH, PB, and DB drafted the manuscript. All authors reviewed the manuscript and agreed to the final version of the manuscript.

## References

[B1] Bernard G. Gagneul D. Alves Dos Santos H. Etienne A. Hilbert J.-L. Rambaud C. (2019). Efficient Genome Editing Using CRISPR/Cas9 Technology in Chicory. *Int. J. Mol. Sci.* 20:1155. 10.3390/ijms20051155 30845784 PMC6429391

[B2] Bogdanović M. Cankar K. Todorović S. Dragicević M. Simonović A. van Houwelingen A. (2019). Tissue specific expression and genomic organization of bitter sesquiterpene lactone biosynthesis in *Cichorium intybus* L. (Asteraceae). *Ind. Crops Prod.* 129 253–260.

[B3] Bouwmeester H. J. Kodde J. Verstappen F. W. Altug I. G. de Kraker J. W. Wallaart T. E. (2002). Isolation and characterization of two germacrene A synthase cDNA clones from chicory. *Plant Physiol.* 129 134–144. 10.1104/pp.001024 12011345 PMC155878

[B4] Cankar K. Bundock P. Sevenier R. Hakkinen S. T. Hakkert J. C. Beekwilder J. (2021). Inactivation of the germacrene A synthase genes by CRISPR/Cas9 eliminates the biosynthesis of sesquiterpene lactones in *Cichorium intybus* L. *Plant Biotechnol. J.* 19 2442–2453. 10.1111/pbi.13670 34270859 PMC8633505

[B5] Cankar K. van Houwelingen A. Bosch D. Sonke T. Bouwmeester H. Beekwilder J. (2011). A chicory cytochrome P450 mono-oxygenase CYP71AV8 for the oxidation of (+)-valencene. *FEBS Lett.* 585 178–182. 10.1016/j.febslet.2010.11.040 21115006

[B6] Cankar K. van Houwelingen A. Goedbloed M. Renirie R. de Jong R. M. Bouwmeester H. (2014). Valencene oxidase CYP706M1 from Alaska cedar (*Callitropsis nootkatensis*). *FEBS Lett.* 588 1001–1007. 10.1016/j.febslet.2014.01.061 24530525

[B7] Chadwick M. Trewin H. Gawthrop F. Wagstaff C. (2013). Sesquiterpenoids lactones: Benefits to plants and people. *Int. J. Mol. Sci.* 14 12780–12805. 10.3390/ijms140612780 23783276 PMC3709812

[B8] De Bruyn C. Ruttink T. Eeckhaut T. Jacobs T. De Keyser E. Goosens A. (2020). Establishment of CRISPR/Cas9 Genome Editing in Witloof (*Cichorium intybus var. foliosum*). *Front. Genome Ed.* 2:604876. 10.3389/fgeed.2020.604876 34713228 PMC8525355

[B9] Deryckere D. Eeckhaut T. Van Huylenbroeck J. Van Bockstaele E. (2012). Low melting point agarose beads as a standard method for plantlet regeneration from protoplasts within the *Cichorium* genus. *Plant Cell Rep.* 31 2261–2269. 10.1007/s00299-012-1335-8 22926032

[B10] Frey M. Klaiber I. Conrad J. Bersch A. Pateraki I. Ro D. K. (2019). Characterization of CYP71AX36 from Sunflower (*Helianthus annuus* L. Asteraceae). *Sci. Rep.* 9:14295. 10.1038/s41598-019-50520-6 PMC6778120 31586110

[B11] Frey M. Schmauder K. Pateraki I. Spring O. (2018). Biosynthesis of Eupatolide-A Metabolic Route for Sesquiterpene Lactone Formation Involving the P450 Enzyme CYP71DD6. *ACS Chem. Biol.* 13 1536–1543. 10.1021/acschembio.8b00126 29758164

[B12] Gietz R. D. Woods R. A. (2002). Transformation of yeast by lithium acetate/single-stranded carrier DNA/polyethylene glycol method. *Methods Enzymol.* 350 87–96. 10.1016/s0076-6879(02)50957-5 12073338

[B13] Gou J. Hao F. Huang C. Kwon M. Chen F. Li C. (2018). Discovery of a non-stereoselective cytochrome P450 catalyzing either 8alpha- or 8beta-hydroxylation of germacrene A acid from the Chinese medicinal plant Inula hupehensis. *Plant J.* 93 92–106. 10.1111/tpj.13760 29086444

[B14] Hall R. D. Riksen-Bruinsma T. Weyens G. J. Rosquin I. J. Denys P. N. Evans I. J. (1996). A high efficiency technique for the generation of transgenic sugar beets from stomatal guard cells. *Nat. Biotechnol.* 14 1133–1138. 10.1038/nbt0996-1133 9631066

[B15] Huber M. Epping J. Schulze Gronover C. Fricke J. Aziz Z. Brillatz T. (2016). A Latex Metabolite Benefits Plant Fitness under Root Herbivore Attack. *PLoS Biol.* 14:e1002332. 10.1371/journal.pbio.1002332 26731567 PMC4701418

[B16] Ikezawa N. Gopfert J. C. Nguyen D. T. Kim S. U. O’Maille P. E. Spring O. (2011). Lettuce costunolide synthase (*CYP71BL2*) and its homolog (*CYP71BL1*) from sunflower catalyze distinct regio- and stereoselective hydroxylations in sesquiterpene lactone metabolism. *J. Biol. Chem.* 286 21601–21611. 10.1074/jbc.M110.216804 21515683 PMC3122218

[B17] Kim D. Y. Choi B. Y. (2019). Costunolide-A Bioactive Sesquiterpene Lactone with Diverse Therapeutic Potential. *Int. J. Mol. Sci.* 20:2926. 10.3390/ijms20122926 31208018 PMC6627852

[B18] Li Q. Wang Z. Xie Y. Hu H. (2020). Antitumor activity and mechanism of costunolide and dehydrocostus lactone: Two natural sesquiterpene lactones from the Asteraceae family. *Biomed. Pharmacother.* 125:109955. 10.1016/j.biopha.2020.109955 32014691

[B19] Liu Q. Beyraghdar Kashkooli A. Manzano D. Pateraki I. Richard L. Kolkman P. (2018). Kauniolide synthase is a P450 with unusual hydroxylation and cyclization-elimination activity. *Nat. Commun.* 9:4657. 10.1038/s41467-018-06565-8 30405138 PMC6220293

[B20] Liu Q. Majdi M. Cankar K. Goedbloed M. Charnikhova T. Verstappen F. W. (2011). Reconstitution of the costunolide biosynthetic pathway in yeast and Nicotiana benthamiana. *PLoS One* 6:e23255. 10.1371/journal.pone.0023255 21858047 PMC3156125

[B21] Liu Q. Manzano D. Tanic N. Pesic M. Bankovic J. Pateraki I. (2014). Elucidation and in planta reconstitution of the parthenolide biosynthetic pathway. *Metab. Eng.* 23 145–153. 10.1016/j.ymben.2014.03.005 24704560

[B22] Matos M. S. Anastacio J. D. Nunes Dos Santos C. (2021). Sesquiterpene Lactones: Promising Natural Compounds to Fight Inflammation. *Pharmaceutics* 13:991. 10.3390/pharmaceutics13070991 34208907 PMC8309091

[B23] Nelson D. R. (2009). The cytochrome p450 homepage. *Hum. Genomics* 4 59–65. 10.1186/1479-7364-4-1-59 19951895 PMC3500189

[B24] Nguyen D. T. Gopfert J. C. Ikezawa N. Macnevin G. Kathiresan M. Conrad J. (2010). Biochemical conservation and evolution of germacrene A oxidase in asteraceae. *J. Biol. Chem.* 285 16588–16598. 10.1074/jbc.M110.111757 20351109 PMC2878029

[B25] Padilla-Gonzalez G. F. dos Santos F. A. Da Costa F. B. (2016). Sesquiterpene Lactones: More Than Protective Plant Compounds With High Toxicity. *Crit. Rev. Plant Sci.* 35 0735–2689. 10.1080/07352689.2016.1145956

[B26] Pompon D. Louerat B. Bronine A. Urban P. (1996). Yeast expression of animal and plant P450s in optimized redox environments. *Methods Enzymol.* 272 51–64. 10.1016/s0076-6879(96)72008-6 8791762

[B27] Prasifka J. R. Spring O. Conrad J. Cook L. W. Palmquist D. E. Foley M. E. (2015). Sesquiterpene Lactone Composition of Wild and Cultivated Sunflowers and Biological Activity against an Insect Pest. *J. Agric. Food Chem.* 63 4042–4049. 10.1021/acs.jafc.5b00362 25853587

[B28] Rao Vadaparthi P. R. Kumar K. Sarma V. U. Hussain Q. A. Babu K. S. (2015). Estimation of Costunolide and Dehydrocostus Lactone in Saussurea lappa and its Polyherbal Formulations followed by their Stability Studies Using HPLC-DAD. *Pharmacogn. Mag.* 11 180–190. 10.4103/0973-1296.149736 25709231 PMC4329622

[B29] Roberfroid M. B. (2002). Functional foods: Concepts and application to inulin and oligofructose. *Br. J. Nutr.* 87 S139–S143. 10.1079/BJNBJN/2002529 12088510

[B30] Roberfroid M. B. (2007). Inulin-type fructans: Functional food ingredients. *J. Nutr.* 137 2493S–2502S. 10.1093/jn/137.11.2493S 17951492

[B31] Sessa R. A. Bennett M. H. Lewis M. J. Mansfield J. W. Beale M. H. (2000). Metabolite profiling of sesquiterpene lactones from Lactuca species. Major latex components are novel oxalate and sulfate conjugates of lactucin and its derivatives. *J. Biol. Chem.* 275 26877–26884. 10.1074/jbc.M000244200 10858433

[B32] Urban P. Mignotte C. Kazmaier M. Delorme F. Pompon D. (1997). Cloning, yeast expression, and characterization of the coupling of two distantly related *Arabidopsis thaliana* NADPH-cytochrome P450 reductases with P450 CYP73A5. *J. Biol. Chem.* 272 19176–19186. 10.1074/jbc.272.31.19176 9235908

[B33] Weber E. Engler C. Gruetzner R. Werner S. Marillonnet S. (2011). A modular cloning system for standardized assembly of multigene constructs. *PLoS One* 6:e16765. 10.1371/journal.pone.0016765 21364738 PMC3041749

[B34] Wesolowska A. Nikiforuk A. Michalska K. Kisiel W. Chojnacka-Wojcik E. (2006). Analgesic and sedative activities of lactucin and some lactucin-like guaianolides in mice. *J. Ethnopharmacol.* 107 254–258. 10.1016/j.jep.2006.03.003 16621374

[B35] Yoo S. D. Cho Y. H. Sheen J. (2007). Arabidopsis mesophyll protoplasts: A versatile cell system for transient gene expression analysis. *Nat. Protoc.* 2 1565–1572. 10.1038/nprot.2007.199 17585298

[B36] Zhang S. Won Y. K. Ong C. N. Shen H. M. (2005). Anti-cancer potential of sesquiterpene lactones: Bioactivity and molecular mechanisms. *Curr. Med. Chem. Anticancer. Agents* 5 239–249. 10.2174/1568011053765976 15992352

