# Supplementary material for: CRISPR/Cas9 targeted inactivation of the kauniolide synthase in chicory results in accumulation of costunolide and its conjugates in taproots
Source: Front Plant Sci. 2022 Aug 29;13:940003. doi: 10.3389/fpls.2022.940003 (PMC9465254; doi:10.3389/fpls.2022.940003)
Supplement: Supplementary file 1 [file Data_Sheet_1.pdf]

Supplementary Figure 1 Protein alignment and phylogenetic tree of the chicory kauniolide synthase genes CiKLS1, CiKLS2 and CiKLS3. A. Phylogenetic tree is shown for chicory kauniolide synthase genes CiKLS1, CiKLS2, CiKLS3 and other cytochrome P450 genes that were described to be involved in STL biosynthesis in Asteraceae family, namely *Lactuca sativa* germacrene A oxidase (LsGAO), *Cichorium intybus* germacrene A oxidase (CiGAO), *Lactuca sativa* costunolide synthase (LsCOS), *Cichorium intybus* costunolide synthase (CiCOS), *Helianthus annuus* germacrene A acid 8β-hydroxylase (HaG8H), *Inula hupehensis* germacrene A acid 8β-hydroxylase (IhG8H), *Tanacetum parthenium* kauniolide synthase (TpKLS), *Tanacetum parthenium* parthenolide synthase (TpPTS), *Tanacetum parthenium* 3β-hydroxylase (Tp3BH), *Helianthus annuus* eupatolide synthase (HaES) and *Helianthus annuus* costunolide 14-hydroxylase (HaC14H). B. Protein alignment of the three chicory KLS genes (CiKLS1, CiKLS2 and CiKLS3) and the *Tanacetum parthenium* kauniolide synthase (TpKLS) is shown. The transmembrane domain and conserved regions of P450 enzymes are indicated. C. Protein sequence identity matrix. D. Nucleotide sequence identity matrix.

A.

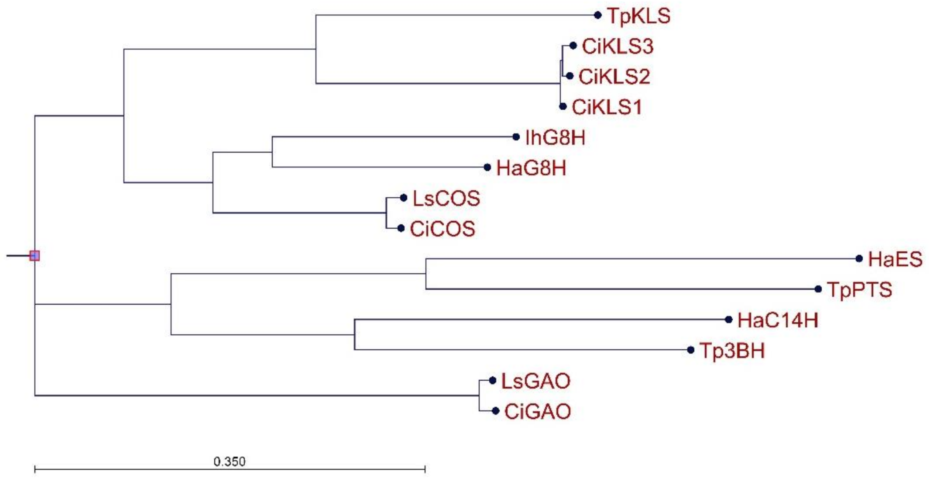

B.

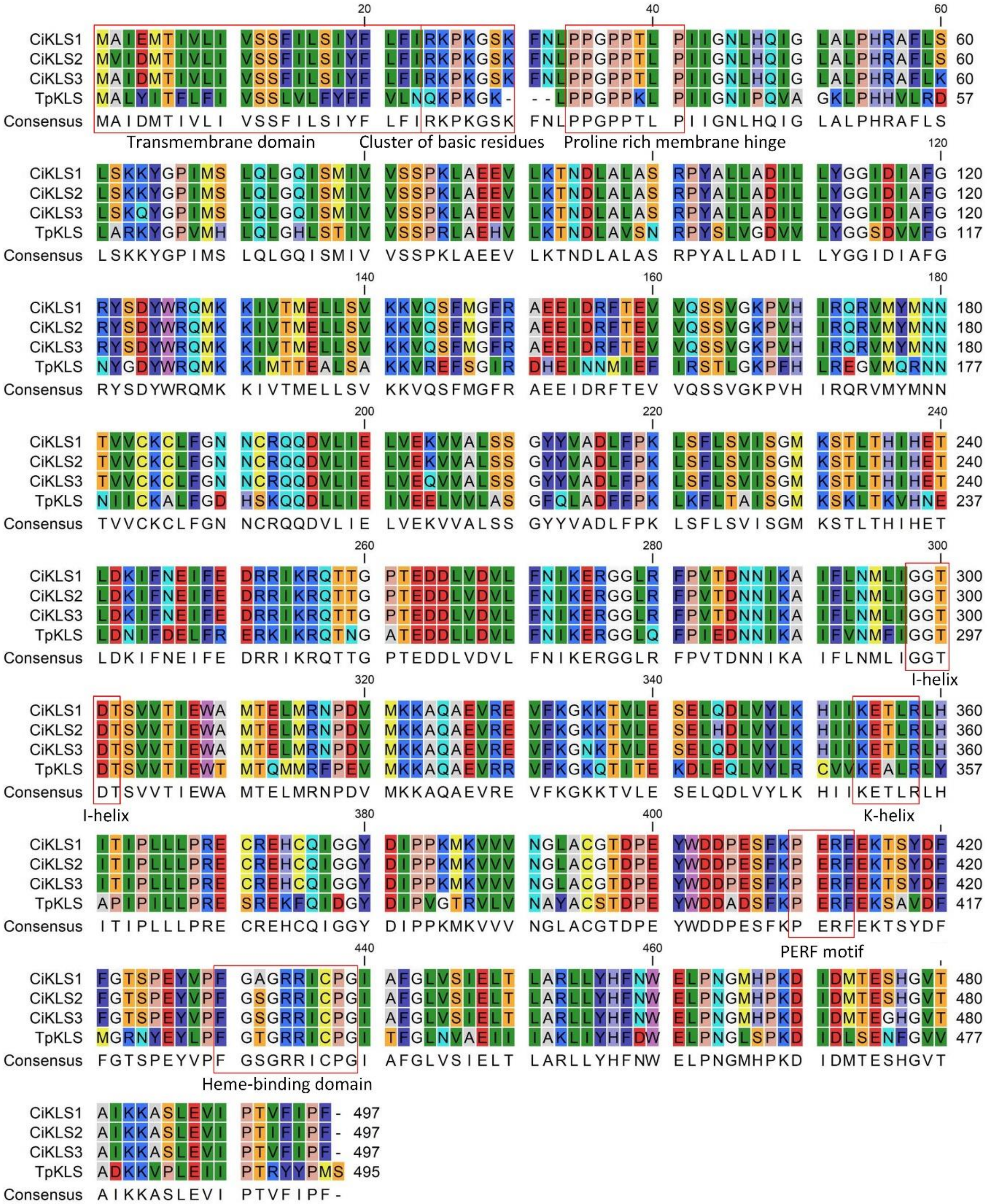
CiKLS1
CiKLS2
CiKLS3
TpKLS
Consensus
Transmembrane domain
Cluster of basic residues
Proline rich membrane hinge
I-helix
K-helix
PERF motif
Heme-binding domain

C.

Percent identity*		Identities**			
		CiKLS1	CiKLS2	CiKLS3	TpKLS
	CiKLS1		492	490	309
	CiKLS2	98.99		489	308
	CiKLS3	98.59	98.39		307
	TpKLS	62.05	61.85	61.65	

*The percentage of overlapping alignment position where the two sequences agree

**The number of overlapping alignment position where the two sequences agree

D.

Percent identity*		Identities**			
		CiKLS1	CiKLS2	CiKLS3	TpKLS
	CiKLS1		1472	1441	1083
	CiKLS2	98.53		1433	1090
	CiKLS3	96.45	95.92		1075
	TpKLS	72.34	72.81	71.81	

*The percentage of overlapping alignment position where the two sequences agree

**The number of overlapping alignment position where the two sequences agree

Supplementary Figure 2 Genomic organization of the KLS genes in chicory. Genes CiKLS1, CiKLS2 and CiKLS3 are co-localized in the chicory genome. (ex1 – exon 1, ex2 – exon 2)

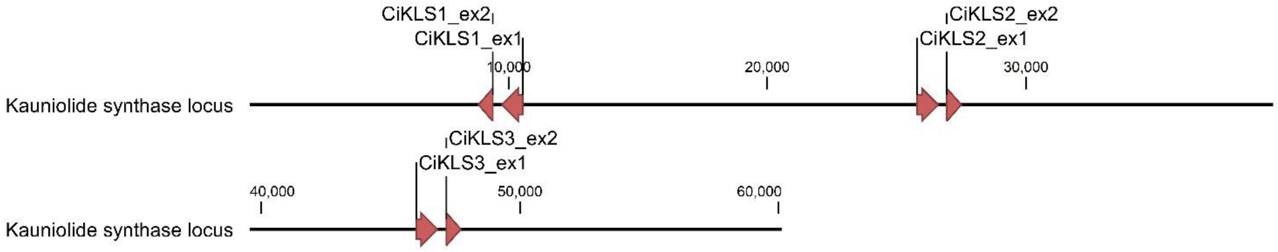

Supplementary Figure 3 Guide position on exon 1 of the chicory kauniolide synthase genes. Shaded areas represent the guide location, PAM sequence is underlined. Guides KLSg2 and KLSg3 target genes CiKLS1 and CiKLS2. Guides KLSg21 and KLSp1 target all three kauniolide synthase genes without mismatches.

```
CiKLS1 GGGTTCCAAGTTTAACCTGCCACCAGGCCCACCAACGCTACCCATCATCGGAAACCTCCATCAGATTGGTTTAGCA
CiKLS2 GGGTTCCAAGTTTAACCTGCCACCAGGCCCACCAACGCTACCCATCATCGGAAACCTCCATCAGATTGGTTTAGCA
CiKLS3 GGGTTCCAAGTTTAACCTGCCACCAGGCCCACCAACGCTACCCATCATCGGAAACCTCCATCAAATTGGTTTAGCA

                                                                       KLSg2

CiKLS1 CTGCCACATCGTGCTTTCCTCTCGTTGTCCAAGAAATACGGCCCCATCATGAGCCTGCAACTCGGCCAGATCTCCA
CiKLS2 CTGCCACATCGTGCTTTCCTCTCGTTGTCCAAGAAATACGGCCCCATCATGAGCCTGCAACTCGGCCAGATCTCCA
CiKLS3 CTGCCACATCGTGCTTTCCTTAAGTTGTCCAAGCAATACGGCCCCATCATGAGCCTGCAACTCGGCCAGATCTCCA
                   KLSg3                                KLSg21

CiKLS1 TGATCGTCGTCTCGTCCCCTAAATTAGCTGAAGAAGTTCTAAAAACCAACGACCTCGCCCTTGCAAGCCGACCATA
CiKLS2 TGATCGTCGTCTCGTCCCCTAAATTAGCTGAAGAAGTTCTAAAAACCAACGACCTCGCCCTTGCAAGCCGACCATA
CiKLS3 TGATCGTCGTCTCGTCCCCTAAATTAGCTGAAGAAGTTCTAAAAACCAACGACCTCGCCCTTGCAAGCCGCCCATA

CiKLS1 TGCACTTCTTGCCGATATTCTGTTGTATGGGGGCATCGACATAGCTTTTGGTCGTTACAGTGATTACTGGCGACAA
CiKLS2 TGCACTTCTTGCCGATATTCTGTTGTATGGGGGCATCGACATAGCTTTTGGTCGTTACAGTGATTACTGGCGACAA
CiKLS3 TGCACTTCTTGCCGATATTCTGTTGTATGGAGGCATCGACATAGCTTTTGGTCGTTATAGTGATTACTGGCGACAA

CiKLS1 ATGAAGAAGATCGTCACCATGGAACTTTTAAGTGTCAAAAAAGTCCAATCCTTCAT
CiKLS2 ATGAAGAAGATCGTCACCATGGAACTTTTAAGTGTCAAAAAAGTCCAATCCTTCAT
CiKLS3 ATGAAGAAGATCGTCACCATGGAACTCTTAAGTGTCAAAAAAGTCCAATCCTTCAT
               KLSp1
```

Supplementary Figure 4 Genotyping mutations in CiKLS genes in chicory protoplast population. Mutations are shown for A. CiKLS1, B. CiKLS2 and C. CiKLS3 gene after plasmid transfection. Mutations shown in panel D are for CiKLS2 using the RNP approach where a single guide was used. The first line for each gene shows the reference WT sequence. The target positions of the guides are underlined and the PAM sites are shown in bold. In the lines below the reference sequences of the most frequently observed mutations are shown with the corresponding mutation frequency.

A. CiKLS1, Total frequency of mutations = 8.4 %

AAA**CCT**CCATCAGATTGGTTTAGCACTG**CCA**CATCGTGCTTTCCTCTCGTTGTCCAAGAAATACGG**CCC**CATCATGAGCCTGCAACTCGGCCA WT

AAACCTCCATCA--CATGAGCCTGCAACTCGGCCA 0.60%

AAACCTCCATCAGATTGGTTTAGCACTGCCACATC-------------------------------------CATGAGCCTGCAACTCGGCCA 0.60%

AAACCTCCA------------AGCACTGCC---CTGCAACTCGGCCA 0.50%

AAACCTCCA**C**TCAGATTGGTTTAGCACTGCCACATC------------------------------------CATGAGCCTGCAACTCGGCCA 0.50%

AAACCTCCAT**T**CAGATTGGTTTAGCACTGCCACATC--TGCAACTCGGCCA 0.47%

AAACCT---------TGGTTTAGCACTGCCACA--CTGCAACTCGGCCA 0.45%

AAACCTCCA**A**TCAGATTGGTTTAGCACTGCCACATC------------------------------------CATGAGCCTGCAACTCGGCCA 0.45%

AAACCTC-------------TAGCACTGCCACAT---GCCTGCAACTCGGCCA 0.37%

AAACCTCCAT---------TTAGCACTGCCACATC-----------TCGTTGTCCAAGAAATACGGCCCCATC-------TGCAACTCGGCCA 0.22%

B. CiKLS2, Total frequency of mutations = 5.5 %

AAA**CCT**CCATCAGATTGGTTTAGCACTG**CCA**CATCGTGCTTTCCTCTCGTTGTCCAAGAAATACGG**CCC**CATCATGAGCCTGCAACTCGGCCA WT

AAACCTCCATCAGATTGGTTTAGCACTGCCACATC---TGCAACTCGGCCA 0.67%

AAACCTCCA**A**TCAG--CATGAGCCTGCAACTCGGCCA 0.65%

AAACCTCCA**A**TCAGATTGGTTTAGCACTGCCACAT--GCAACTCGGCCA 0.44%

AAACCTCCATCAGATTGGTTTAGCACTGCCACAT--CAACTCGGCCA 0.37%

AAACCTC--------------------------TCGT-------------TGTCCAAGAAATACGGCCCCATCATGAGCCTGCAACTCGGCCA 0.38%

AAACCTCCAT**T**CGAATTGGTTTAGCACTGCCACATC------------------------------------CATGAGCCTGCAACTCGGCCA 0.36%

C. CiKLS3, Total frequency of mutations = 3.4 %

AAACCTCCATCAAATTGGTTTAGCACTGCCACATCGTGCTTTCCTTAAGTTGTCCAAGAAATACGG**CCC**CATCATGAGCCTGCAACTCGGCCA WT

AAACCTCCATCAAATTGGTTTAGCACTGCCACATCGTGCTTTCCTTAAGTTGTCCAAGAAATACGGCCCCATC-------TGCAACTCGGCCA 0.67%

AAACCTCCATCAAATTGGTTTAGCACTGCCACATCGTGCTTTCCTTAAGTTGTCCAAGAAATACGGCCCCAT--------------TCGGCCA 0.50%

AAACCTCCATCAAATTGGTTTAGCACTGCCACATCGTGCTTTCCTTAAGTTGTCCAAGAAATACGGCC------------TGCAACTCGGCCA 0.22%

AAACCTCCATCAAATTGGTTTAGCACTGCCACATCGTGCTTTCCTTAAGTTGTCCAAGAAATACGGCCC--------------AACTCGGCCA 0.21%

D. CiKLS2, Total frequency of mutations = 3%

ACAAATGAAGAAGATCGTCACCA**TGG**AAC

ACAAATGAAGAAGATCGTCAA*CCATGG*AAC 1.2%

ACAAATGAAGAAGATC-----*CATGG*AAC 0.2%

Supplementary Figure 5 Terpene profiling of the leaves of gene edited chicory plants by LC-Orbitrap-FTMS. Peak area of lactucin (L), lactucopicrin (LP), 8-deoxylactucin (8-DOL), lactucin 15-oxalate (Lox), 8-deoxylactucin 15-oxalate (8-DOLox) and lactucopicrin 15-oxalate (LPox) is shown for first (A), second (B) and third (C) round of screening of genome edited plants (lines marked with A – plasmid transfection, lines marked with B – RNP transfection) in comparison to wild-type chicory plants (WT). In total 24 from the 45 analysed chicory plants show nearly complete elimination of STLs.

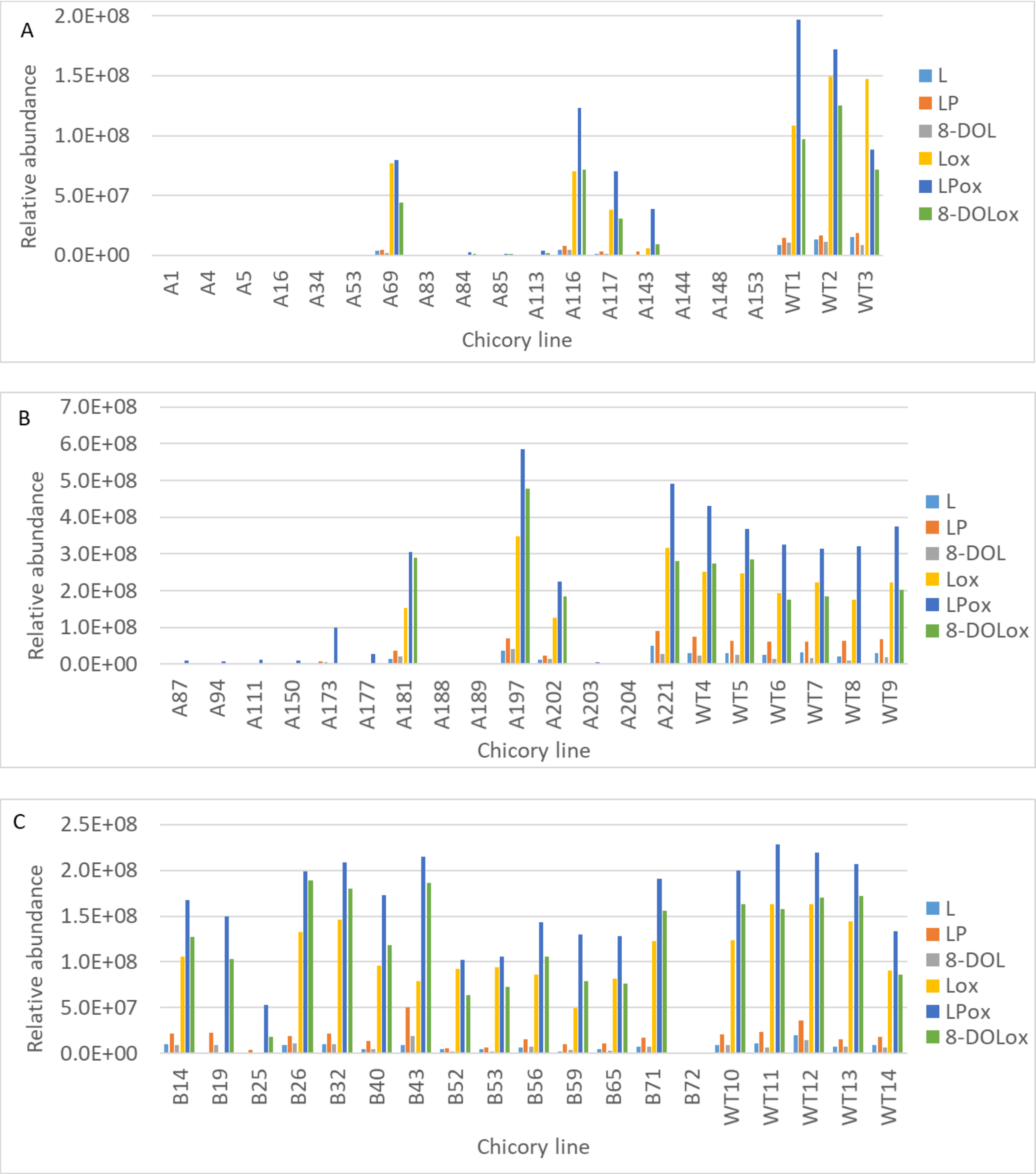

Supplementary Figure 6 LC-Orbitrap-FTMS chromatograms and mass spectra of terpenes produced in the chicory root. A. Terpene profile of chicory taproot of genome edited line A83 compared to WT taproot. Chromatograms are shown at the same scale. Peak 1 – costunolide-cysteine, Peak 2 – costunolide-glutathione, Peak 3 – unknown costunolide conjugate with mass of $[M+H]^+ = 441.15692$, Peak 4 – costunolide, Peak 5 – lactucin, Peak 6 – lactucin 15-oxalate, Peak 7 - 8-deoxylactucin, Peak 8 - 8-deoxylactucin 15-oxalate, peak 9 – lactucopicrin, Peak 10 - lactucopicrin 15-oxalate. B. Mass spectra of peaks 1-10 and authentic standards of lactucin, lactucopicrin and costunolide.

A.

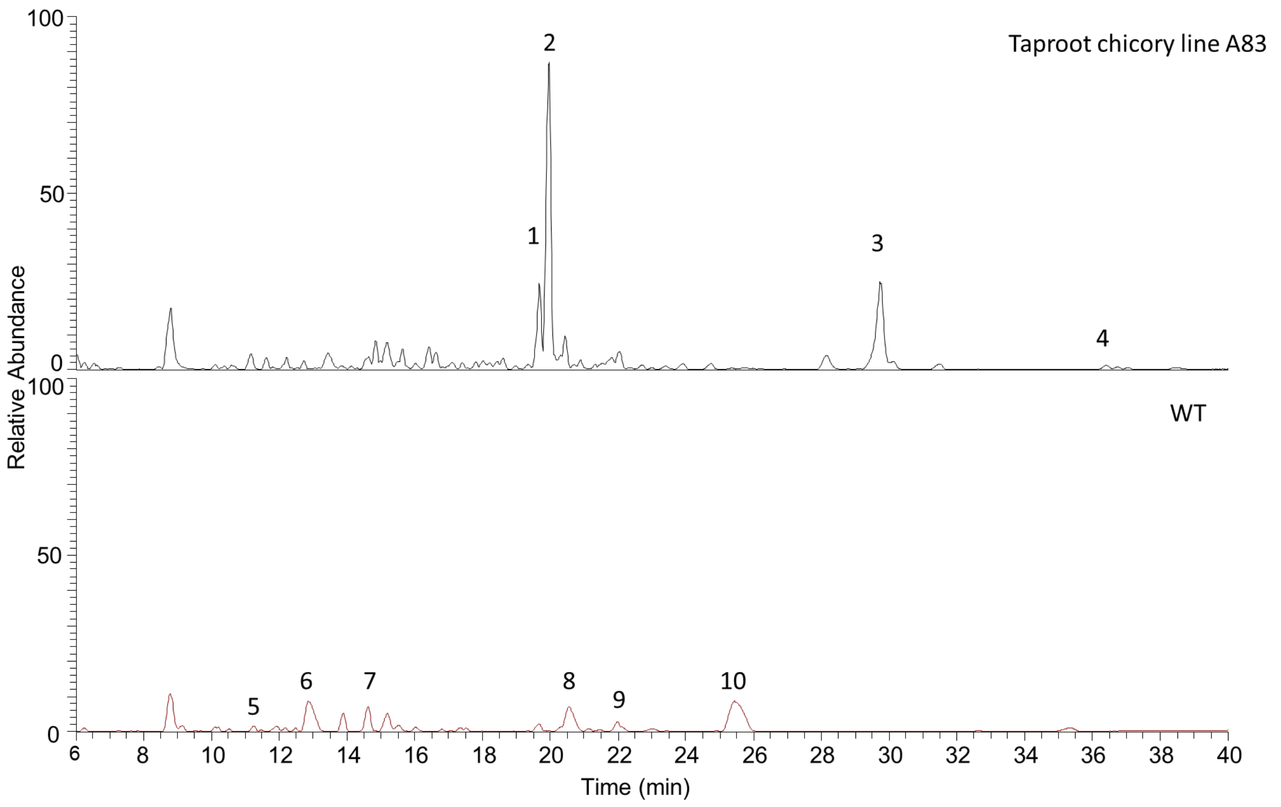

B.

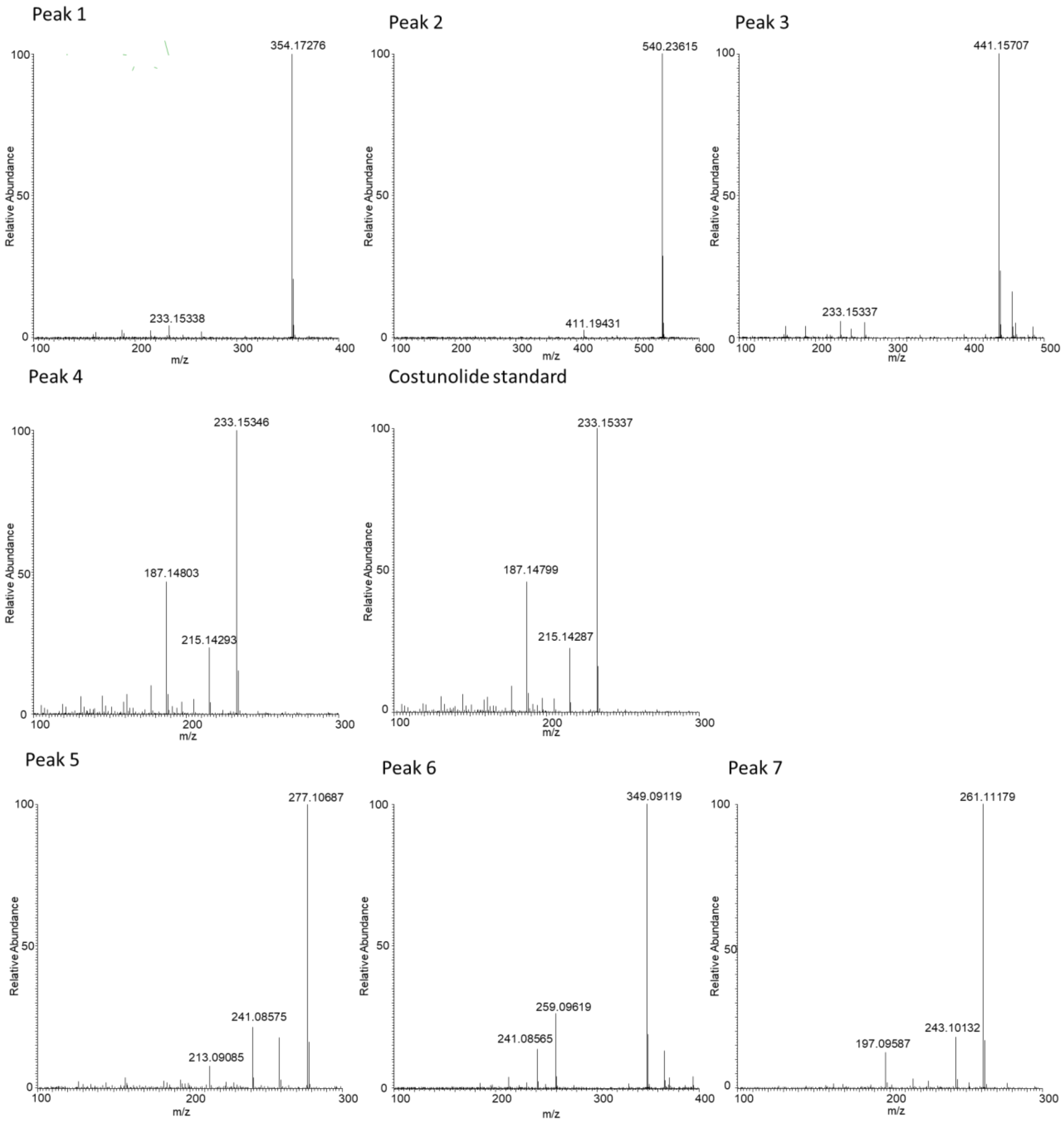
Peak 1
354.17276
233.15338
Peak 2
540.23615
411.19431
Peak 3
441.15707
233.15337
Peak 4
233.15346
187.14803
215.14293
Costunolide standard
233.15337
187.14799
215.14287
Peak 5
277.10687
241.08575
213.09085
Peak 6
349.09119
259.09619
241.08565
Peak 7
261.11179
243.10132
197.09587
Relative Abundance
m/z

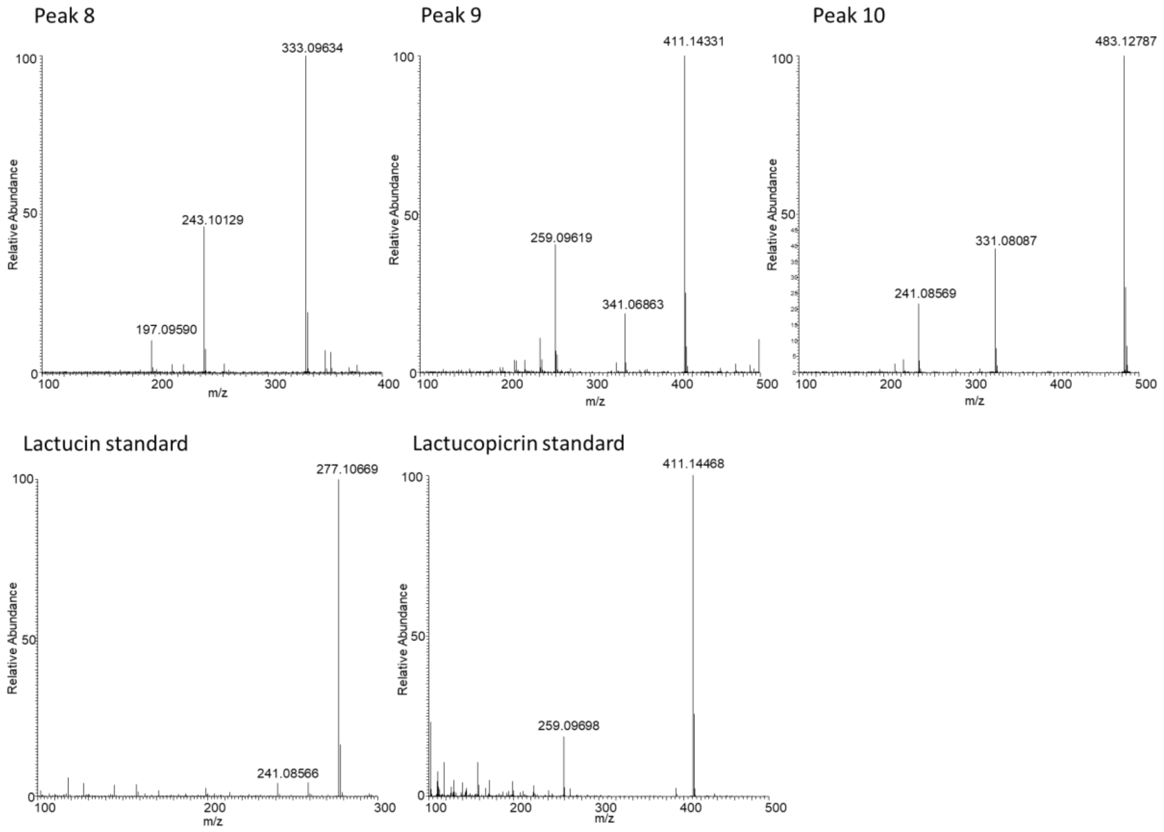
Peak 8
333.09634
243.10129
197.09590
Relative Abundance
m/z
Peak 9
411.14331
259.09619
341.06863
Relative Abundance
m/z
Peak 10
483.12787
331.08087
241.08569
Relative Abundance
m/z
Lactucin standard
277.10669
241.08566
Relative Abundance
m/z
Lactucopicrin standard
411.14468
259.09698
Relative Abundance
m/z

Supplementary Figure 7 GC-MS chromatogram of chicory taproot extract. Extract of chicory line A83 is shown in comparison to a WT chicory root extract. Peak 1 – germacrene A acid, Mw = 234.3, Peak 2 – costunolide, Mw =232.3.

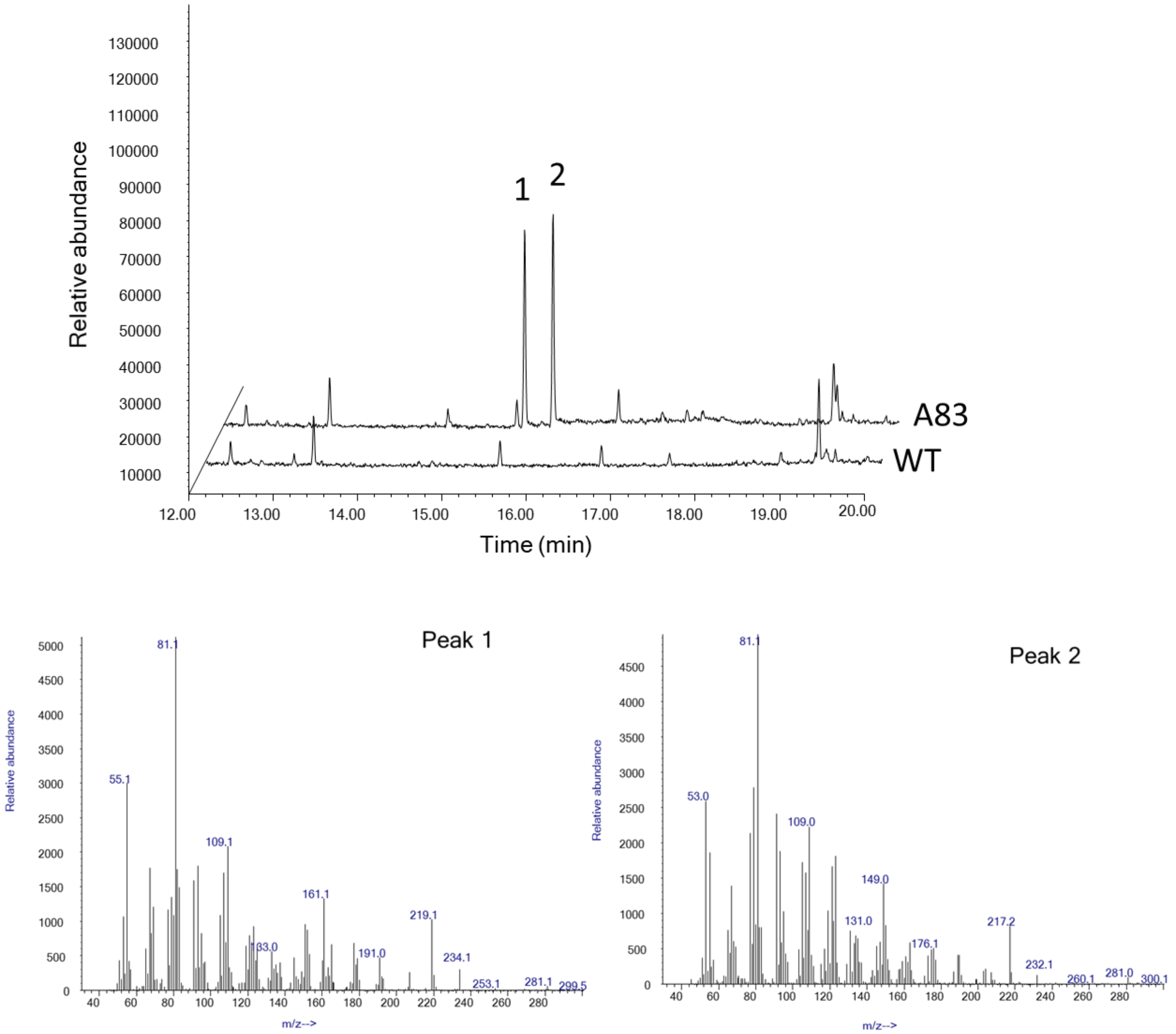

Supplementary Table 1 Guide and primer sequences used in this study

Name	Sequence	Target gene	Purpose
KLSg2	GTGCTAAACCAATCTGATGG	CiKLS1, CiKLS2	Guide RNA
KLSg3	AACGAGAGGAAAGCACGATG	CiKLS1, CiKLS2	Guide RNA
KLSg21	CGAGTTGCAGGCTCATGATG	CiKLS1, CiKLS2, CiKLS3	Guide RNA
KLSp1	AATGAAGAAGATCGTCACCA	CiKLS1, CiKLS2, CiKLS3	Guide RNA
CiKLS1-Fw1	TCCTACACCACTTCCCCAGTTCC	CiKLS1	genotyping protoplasts
CiKLS2-Fw1	CACCATTGAGTAAGTAACAATGGTCATCGA	CiKLS2	genotyping protoplasts
CiKLS3-Fw1	TGTACACCATTCAGTCATTCAGTGTGTAAC	CiKLS3	genotyping protoplasts
CiKLS1-Rev1	ATAATTGATGGGAGTAGTGACGTACCAGA	CiKLS1	genotyping protoplasts
CiKLS2+3-Rev1	ACAACATAAGCAGAGTATGGACAAACCAGA	CiKLS2, CiKLS3	genotyping protoplasts
CiKLS1-Fw2	ATTCTCCTACACCACTTCCCC	CiKLS1,CiKLS2, CiKLS3	genotyping protoplasts
CiKLS1-Rev2	CCACTCGATAATGCCACCAC	CiKLS1	genotyping protoplasts
CiKLS2-Fw2	ACCCGTCTTTCTAATACACCA	CiKLS2	genotyping protoplasts
CiKLS2-Rev2	GCCACCACTTTTTCCACC	CiKLS1, CiKLS2	genotyping protoplasts
CiKLS3-Fw2	GCCATCTTTCTGTACACCATTC	CiKLS3	genotyping protoplasts
CiKLS3-Rev2	ACTCGATAATGCCACCACT	CiKLS1,CiKLS2,CiKLS3	genotyping protoplasts
CiKLSnestF1	ACTCTTTCCCTACACGACGCTCTTCCGATCT GGCCCACCAACGCTACCCAT	CiKLS1, CiKLS2, CiKLS3	nested PCR genotyping protoplasts
CiKLSnestR1	TGGAGTTCAGACGTGTGCTCTTCCGATCTC GAGACGACGATCATGGAGA	CiKLS1, CiKLS2, CiKLS3	nested PCR genotyping protoplasts
CiKLSnestF2	ACTCTTTCCCTACACGACGCTCTTCCGATCT TTTGGTCGTTACAGTGATTA	CiKLS1, CiKLS2, CiKLS3	nested PCR genotyping protoplasts
CiKLSnestR2	TGGAGTTCAGACGTGTGCTCTTCCGATCTA AAACCCATGAAGGATTGGA	CiKLS1, CiKLS2, CiKLS3	nested PCR genotyping protoplasts
CiKLS-all-Fw	CTCTCCATCTACTTCCTCTTCATCAGAA	CiKLS1, CiKLS2, CiKLS3	common primer genotyping calli/shoots
CiKLS-all-Rev	CTTAACTTAGGAAACAGGTCTGCAACA	CiKLS1, CiKLS2, CiKLS3	common primer genotyping calli/shoots
aCas9 Fw2	CTACCAGACTCAAGAGAACCGCTAG	Cas9	Cas9 detection
aCas9 Rev2	GTGGTGCTCATCGTATCTCTTGATCA	Cas9	Cas9 detection

Supplementary Table 2 Genotyping of the CiKLS1, CiKLS2 and CiKLS3 genes in 9 gene edited lines.

Chicory line	Genotype CiKS1	Genotype CiKS2	Genotype CiKS3
A1	**Homozygous**<br>68nt del (g2-g21)	**Homozygous**<br>114nt ins (g3)	**Homozygous**<br>9nt del (g21)
A5	**Homozygous**<br>72nt del (g2-g21)	**Bi-allelic**<br>72nt del (g2-g21) *and*<br>16nt ins (g2), 42nt del (g3-g21)	**Homozygous**<br>5nt del (g21)
A16	**Homozygous**<br>38nt del (g3-g21)	**Homozygous**<br>T-ins (g2), 47nt del (g3-g21)	**WT**
A34	**Homozygous**<br>A-ins (g2), 13nt del (g3), 7nt del (g21)	**Bi-allelic**<br>5nt del (g2), 49nt del (g3-g21) *and*<br>70nt del(g2-g21)	**Bi-allelic**<br>6nt del (g21)<br>68nt ins (g21)
A83	**Homozygous**<br>3nt del (g2), 44nt del (g3-g21)	**Bi-allelic**<br>3nt del (g2), 44nt del (g3-g21) *and*<br>A-ins (g2), 46nt del (g3-g21)	**Bi-allelic**<br>A-ins (g21) *and*<br>10nt del (g21)
A144	**Homozygous**<br>Tins (g2), 27nt ins	**Homozygous**<br>40nt del (g3-g21)	**WT**
A148	**Bi-allelic**<br>9nt del (g2), 38nt del (g3-g21) *and*<br>41nt del (g2-g3), 7nt del (g21)	**Bi-allelic**<br>A-ins (g2), 38nt del (g3-g21) *and*<br>314nt ins (g2), C-ins (g3), 7nt del (g21)	**Heterozygous**<br>6nt del (g21) *and*<br>WT
A153	**No PCR fragment obtained**	**Bi-allelic**<br>38nt del (g3-g21) *and*<br>62nt del (g2-g21)	**WT**
B72	**Homozygous**<br>A-ins (p1) / A-ins (p1)	**Heterozygous**<br>A-ins (p1) / A-del (p1)	**Homozygous**<br>A-ins (p1) / A-ins (p1)
